# Heterogeneous pseudobulk simulation enables realistic benchmarking of cell-type deconvolution methods

**DOI:** 10.1186/s13059-024-03292-w

**Published:** 2024-07-01

**Authors:** Mengying Hu, Maria Chikina

**Affiliations:** 1 Department of Computational and Systems Biology, University of Pittsburgh, Pittsburgh, USA; 2 Joint Carnegie Mellon - University of Pittsburgh Computational Biology PhD Program, University of Pittsburgh, Pittsburgh, USA

**Keywords:** Deconvolution, Benchmark, Bulk simulation

## Abstract

**Background:**

Computational cell type deconvolution enables the estimation of cell type abundance from bulk tissues and is important for understanding tissue microenviroment, especially in tumor tissues. With rapid development of deconvolution methods, many benchmarking studies have been published aiming for a comprehensive evaluation for these methods. Benchmarking studies rely on cell-type resolved single-cell RNA-seq data to create simulated pseudobulk datasets by adding individual cells-types in controlled proportions.

**Results:**

In our work, we show that the standard application of this approach, which uses randomly selected single cells, regardless of the intrinsic difference between them, generates synthetic bulk expression values that lack appropriate biological variance. We demonstrate why and how the current bulk simulation pipeline with random cells is unrealistic and propose a heterogeneous simulation strategy as a solution. The heterogeneously simulated bulk samples match up with the variance observed in real bulk datasets and therefore provide concrete benefits for benchmarking in several ways. We demonstrate that conceptual classes of deconvolution methods differ dramatically in their robustness to heterogeneity with reference-free methods performing particularly poorly. For regression-based methods, the heterogeneous simulation provides an explicit framework to disentangle the contributions of reference construction and regression methods to performance. Finally, we perform an extensive benchmark of diverse methods across eight different datasets and find BayesPrism and a hybrid MuSiC/CIBERSORTx approach to be the top performers.

**Conclusions:**

Our heterogeneous bulk simulation method and the entire benchmarking framework is implemented in a user friendly package https://github.com/humengying0907/deconvBenchmarking and https://doi.org/10.5281/zenodo.8206516, enabling further developments in deconvolution methods.

**Supplementary Information:**

The online version contains supplementary material available at 10.1186/s13059-024-03292-w.

## Background

Bulk RNA-sequencing experiments reveal average gene expression values for all cells present in a sample mixture. Computational deconvolution methods separate the mixed signals from the aggregated expression and provide estimation of cellular components without physical isolations. The inferred cellular proportions are important to understand the ecosystem of the tissue and can be used as covariates in differential expression, reducing false positives and false negatives [1, 2]. Moreover, for heterogeneous bulk samples like tumor [3], deconvolution enables identification and quantification of the infiltrating immune populations, which provides rich prognostic values and can guide targeted therapy (e.g., in immunotherapy) [4–8].

Numerous deconvolution methodologies have been developed (see [1], for review), aiming at estimation of cell-type abundance from bulk transcriptomic data. Depending on if and how priori knowledge used, these methods can be broadly classified into four categories: regression-based, marker-based, and reference-free methods. Regression-based methods require an expression matrix as input, which consists of a cell type-specific expression profile for selected genes. These methods then solve the deconvolution as a regression problem. A comprehensive evaluation of factors involved in regression-based methods, like data transformation, normalization, and regression algorithms, can be found elsewhere [9]. Marker-based methods require a set of genes that characterize the expression patterns in different cell types and return either an enrichment score [10] that is unitless or abundance estimates [11, 12].

Reference-free methods [12–14] are completely unsupervised and do not require any prior knowledge as input. Such methods are based on finding a simplex, which is a geometric data structure expected under ideal mixture proportion scenarios. Finally, we note that a recent advance in deconvolution uses a Bayesian framework that relies on a reference matrix but uses it in a way that is distinct from reference based approaches [15].

Rapid development of deconvolution methodologies now raises another challenge of evaluating their performance across diverse realistic settings. Many benchmarking studies have been undertaken to meet this demand [2, 9, 16, 17]. Regardless of the focus of their evaluation, all benchmarking efforts rely on datasets with known ground truth. To acquire such data, one traditional approach involves using real bulk data with paired cell type fraction information, which can be derived from fluorescence-activated cell sorting (FACS) or immunohistochemistry (IHC) staining [17, 18]. However, this approach is restricted by the extensive experimental labor and limited sample availability, making it less practical for large-scale benchmarking studies. An alternative approach is computational mixing where purified expressions of different cell-types are mixed in controlled proportions [10, 19, 20]. While the purely computational strategy can generate large datasets, this approach has the clear limitation that it makes the strong assumption that proportion variation and random noise are the only source of variance in the data.

Increasing availability of single-cell data [21] offers the opportunity to create more realistic simulations. Instead of computational mixing of pure expression states, individual single cell profiles are added together in controlled proportions [9, 17, 18, 22]. This has the explicit advantage over pure computational mixing as it introduces more variations in the simulated samples. However, as we will show in this work, while this approach has rapidly become the standard method for bulk simulation, the problem with unrealistic biological variance is only partially resolved. To simulate data compatible with bulk measurements, a large number of cells (typically hundreds) are added for each simulated sample. As such the pure cell type-specific expression in each sample, while not exactly identical, tends towards the global mean of that cell type in the source scRNA data, enforcing the unrealistic assumption that there is no systematic variation beyond cell-type proportions. One possible solution is to take into account intra-sample heterogeneity in the simulated bulk mixtures. In Chu et al.’s [15] study, they created such simulated bulk mixtures by restricting that the malignant cells aggregated to form a simulated bulk sample originating from the same biological sample. Dong et al. [23] and Menden et al. [24] implemented a simulation strategy that involved repetitively sub-sampling cells from the same patient, ensuring proper inter-sample heterogeneity. However, there is currently no general evaluation of how heterogeneity affects the deconvolution results, as compared with bulk simulation using random cells.

In this study, we introduce a novel heterogeneous simulation approach that aims at capturing accurate biological variance. Through systematic comparison of these simulation methods, we demonstrated that bulk simulation methods using random cells do not reflect realistic biological heterogeneity while our newly proposed approach does. Leveraging the varying heterogeneity levels in the simulated bulk samples, we provided an in-depth comparison of different categories of deconvolution methods using our systematic benchmarking frameworks (Additional file 1: Fig. S1), aiming to elucidate the impact of heterogeneity on the results. By summarizing deconvolution performance across experimental repeats, we found that introducing biological heterogeneity has a notable effect on the deconvolution results, with reference-free methods being most affected. Our study can guide researchers in choosing the most appropriate deconvolution methods, and the highly realistic simulation framework we proposed can facilitate further methodological development.

## Results

### Exploring biological variance in simulated bulk data: influence of different simulation strategies

In previous benchmarking studies [2, 9, 17], the evaluation of deconvolution performance relied on simulated bulk expression using predefined cell-type fractions as ground truth. These studies employed a “homogeneous” simulation approach, where single-cell profiles from single-cell data were combined randomly within each cell type and aggregated in proportions. However, this approach only accounts for cell-type proportion level variance and overlooks other sources of biological variance.

To address this limitation, we explored alternative simulation methods aiming at introducing more biological variance within the simulated samples. We proposed a “heterogeneous” simulation setting where cells used to compose the cell type components of a simulated bulk sample are constrained to come from the same biological samples (Additional file 1: Fig. S2), thus capturing the sample-level heterogeneity [25, 26]. We also considered a less “heterogeneous” setting where only malignant cells are originated from the same sample in the simulation, which we referred to as “semi-heterogeneous” simulation, inspired from Chu’s benchmarking work [15].

We postulated that these three simulation methods—homogeneous, semi-heterogeneous, and heterogeneous—will inherently produce samples with distinct levels of variance, reflecting varied capacity to mimic real biological complexity. To test this, we applied these simulation strategies on four distinct single-cell datasets (Table 1, Additional file 2: Table S1), resulting in a total of 12 simulated expression profiles for evaluation. For cell type fraction simulation, we adopted a beta distribution-based strategy, allowing the mean and variances of each cell-type fractions to be approximately matched to those of real data (see the “Methods” section; Additional file 1: Fig. S3). To create baseline bulk expression for variance comparison, we aggregated single cells from the same patients and used them as approximations of real bulk samples (see the “Methods” section). We also collected expression profiles from the TCGA datasets [27] when the relevant tumor type is available. A detailed description of bulk datasets used in variance comparison can be found in Additional file 2: Table S2.

**Table 1:** Single-cell datasets used in benchmarking

Dataset	Tumor type	# of cells	Publication	Data
Puram2017_HNSCC^a^	HNSCC	5901	Puram et al. 2017 [25]	[28]
Tirosh2016_SKCM^a^	SKCM	4645	Tirosh et al. 2016 [29]	[30]
Riemondy2022_MB^a^	MB	39,946	Riemondy et al. 2022 [31]	[32]
Jerby_Arnon2018_SKCM^a^	SKCM	7186	Jerby-Arnon et al. 2018 [33]	[34]
Lee2020_CRC	CRC	21,657	Lee et al. 2020 [35]	[36]
Qian2020_BRCA	BRCA	16, 537	Qian et al. 2020 [37]	[38]
Kim2020_LUAD	LUAD	32,493	Kim et al. 2020 [39]	[40]
Izar2020_OV	OV	10,788	Izar et al. 2020 [41]	[42]

*HNSCC* head and neck squamous cell carcinomas, *SKCM* skin cutaneous melanoma, *MB* medulloblastoma, *CRC* colorectal cancer, *BRCA* breast cancer, *LUAD* lung adenocarcinoma, *OV* ovarian cancer

^a^scRNA datasets marked with a superscript (a) are also utilized for simulation strategy comparisons

**Fig. 1:**
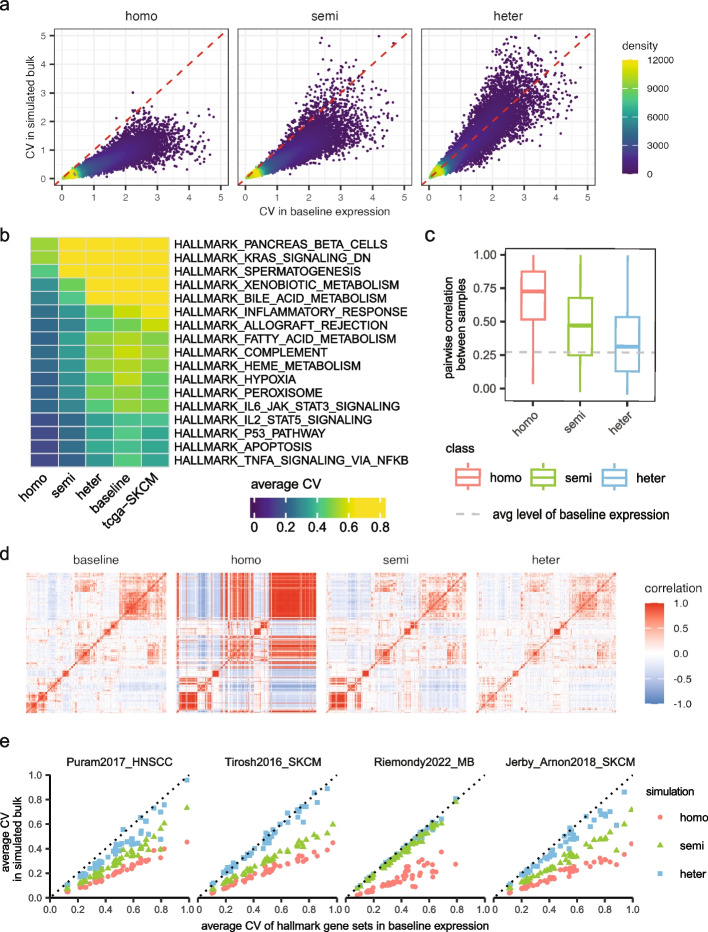
Comparison of different bulk simulation strategies. **a** Scatter plots comparing coefficient of variation (CV) for all genes between the simulated samples and baseline expression. **b** Heatmap comparing average CV of genes from different biological pathways. **c** Boxplot comparing pairwise correlations between simulated bulk samples, with the dashed line indicating the average pairwise correlation in baseline expression. **d** Heatmaps showing gene correlations in bulk samples, using the top 300 most variable genes from the baseline expression. **e** Scatter plots comparing variance of 50 hallmark gene sets between simulated and baseline bulk expression, with each dot representing the averaged CV of genes from a hallmark gene set. In **a**–**d**, all simulated datasets and baseline expression are derived from the Jerby_Arnon2018_SKCM dataset. Throughout the comparison, each simulated dataset comprises 100 simulated samples, and baseline expression is generated by aggregating single-cell expression from the same biological sample, serving as an approximation to real bulk samples

We illustrate our framework with bulk data simulated from Jerby_Arnon2018_SKCM. Utilizing the coefficient of variation (CV) of gene expressions as a measure of intra-sample variance, we first compared gene-level CV between simulated bulk samples and baseline bulk expression. Our findings revealed that the heter-simulated bulk samples exhibited variance closely aligned with that of actual bulk samples, while the homo-simulated samples displayed generally lower variability and semi-heter simulated samples failing between (Fig. 1a). Summarized gene-CV at pathway levels [43] further confirmed this finding, and by extending the CV analysis to include real TCGA bulk samples from the same tumor type, we showed that heter-simulated samples retained proper biological variance compared with real bulk samples (Fig. 1b).

Moving forward, we calculated pairwise correlations between the simulated samples to gain further insight into biological variance (Fig. 1c). The distribution of pairwise correlations across different simulation settings revealed distinct variance levels, with homo-simulated samples showing the highest pairwise correlations, and the heter-simulated samples exhibiting the least pairwise similarities, aligning more closely with real-world settings. Moreover, as we visualized pairwise correlation between genes under different simulation settings, we found that the heter-simulated samples effectively captured proper gene correlations (Fig. 1d). They maintained appropriate gene clusters, similar to those seen in baseline bulk expression, and demonstrated reasonable coefficient correlations. In contrast, homo-simulated samples resulted in false-positive gene clustering structures and spuriously high gene correlations. We note that in this comparison we only focused on the top 300 most variant genes due to the immense scale of the gene-gene correlation matrix; the same patterns hold when specifically considering cell-type marker genes as well (Additional file 1: Fig. S4, Additional file 2: Table S3).

In Fig. 1e, we extended the variance comparison to all datasets by comparing pathway-level variance in simulated samples against real bulk expressions. Our results showed that across each dataset, the heter-simulated samples closely mirrored the actual variance observed in real data, exhibiting pathway-level CVs that align closely with those of real bulk samples, whereas the homo-simulated samples are in general less variable. We note that for bulk data simulated from Riemondy2022_MB dataset, the semi-simulated and heter-simulated samples show little difference with respect to pathway variances; this is because the simulated MB samples are mainly dominated by malignant cells, making it less distinguishable between two strategies.

In addition to the three simulation methods mentioned above, we also investigated four additional bulk simulation approaches: the “favilaco” method [9], the “immunedeconv” method, [17], the “SCDC” method [23], and one “heterogeneous” simulation method that does not rely on single-cell sampleID information, which we referred to as the “heterogeneous-sampleIDfree” method (see the “Methods” section). These expanded methodologies, along with our original simulation strategies, have been integrated into our published package, offering a comprehensive toolkit for bulk simulations.

An extensive comparison of all seven simulation methods is detailed in Additional file 1: Fig. S4-S7. Our analysis revealed that the “heterogeneous-sampleIDfree” method successfully maintained comparable variance without the constraints of sample ID dependency, offering a significant advantage for single-cell datasets where sample IDs may be limited or absent. Conversely, the other two methods, “immunedeconv” and “favilaco,” which were originally implemented for deconvolution benchmarking studies [9, 17], exhibited a notable lack of variance in simulated samples. Finally, the “SCDC” method [23], although it achieved variance comparable to that of real bulk samples, carries the risk of generating empty gene expression values. This is due to its reliance on repeated sub-sampling from the same patient, which can easily suffer from the sparsity issue in single cells [44] when aggregating over only a limited number of cells.

Together, our results suggested that different simulation strategies result in simulated bulk samples with diverse levels of biological variance. Specially, stepping through homogeneous, semi-heterogeneous, and heterogeneous simulation, the heterogeneity level inside samples is increasing with the final heterogeneous simulation closely retaining the characteristics observed in real bulk samples.

### Bulk simulation using random cells ignores heterogeneity within constituent cell types

Heterogeneity of tumors between different patients with the same tumor type has long been recognized [45]. Despite similar histological appearance, different patients can have intrinsically different genomic landscapes. In clinical practice, this heterogeneity motivates molecular subtyping and enables personalized treatment protocols [46, 47]. Retaining biological heterogeneity within simulated bulk samples is essential for realistic bulk simulation.

**Fig. 2:**
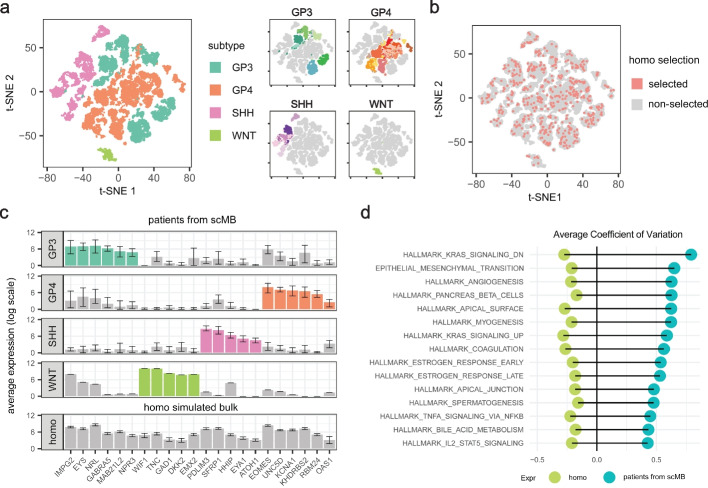
Bulk simulation using random cells failed to retain intra-tumor variations. **a** tSNE plot of *n *= 31,823 malignant cells from 28 medulloblastoma patients in Riemondy2022_MB dataset, colored by MB subtypes (left) and patient ID (right). **b** tSNE plot showing that 500 randomly selected malignant cells are evenly dispersed among 31,823 malignant cells. **c** Barplot showing the average expression levels of 22 MB subtype-specific genes in different groups of samples, colored by different MB subtypes. Upper panel: average expression levels of these genes across different MB subtype patients from Riemondy2022_MB; bottom panel: average expression values in 100 homogeneous simulated bulk samples, with error bars indicating the 10th and 90th quantiles. **d** Diverging bar plot comparing variance of hallmark gene-sets between homo-simulated and patient-specific bulk expression from Riemondy2022_MB, with patient-specific expression defined as the aggregated expression of single cells from the same patient

To illustrate the limitation of bulk simulation using randomly selected cells (namely homogeneous simulation), we considered a simulation setup using single cell Medulloblastoma (MB) dataset from Riemondy et al. [31]. Medulloblastoma is a well-recognized heterogeneous brain cancer with four distinct subtypes based on genetic characteristics: WNT, SHH, Group 3, and Group 4 [48, 49]. Analyzing the tSNE clustering of malignant populations (Fig. 2a), we found that cells from the same subtype predominantly clustered together, revealing marked differences between subtypes. Additionally, within each subgroup, further patient-specific heterogeneity was observed. We note that such intra-heterogeneity of malignant cells extends beyond this scRNA dataset and is also found in other tumor types (Additional file 1: Fig. S8). Additionally, beyond just malignant populations, non-malignant cells may also exhibit diverse patterns across different samples (Additional file 1: Fig. S9). Together, these findings suggested even among cells identified as the same cell type, intra-tumor heterogeneity is not uncommon.

Despite this prevalent intra-heterogeneity, random selection of cells results in an evenly distributed selection of cells (Fig. 2b), and such selection, if performed repeatedly, will create a homogeneous expression profile with low variance. In Fig. 2c, we simulated a bulk expression dataset in this manner and compared it to actual MB patient profiles (Additional file 2: Table S2). By analyzing the expression levels of 22 MB-subtype specific genes (Additional file 2: Table S4) [46], we observed distinct expression patterns across different MB subtypes in real patient profiles, whereas the simulated samples exhibited minimal heterogeneity in expression values of these genes, with the 10% and 90% expression quantiles fluctuate around the average level.

Moreover, analyzing the variation at the pathway level using the hallmark genes [43], we found that systematic pathway-level variance is pervasive in real data but is not recapitulated in the random cell simulation (Fig. 2d). Together, these results suggested that bulk simulation methods employing random single-cell selection overlook meaningful biological variability, resulting in a “homogeneous” profile characterized by low variance.

### Benchmarking framework

To systematically evaluate the performance of different categories of deconvolution methods and examine how different bulk simulation strategies will impact the performance, we designed a benchmarking framework as depicted in Additional file 1: Fig. S1. The deconvolution methods we included span four categories of deconvolution methodologies as mentioned in previous sections: reference-free, regression-based, marker-based, and Bayesian method.

For reference-free methods, we selected debCAM (referred to as CAMfree) [12] and linseed [14]. For regression-based methods, we included five regression algorithms that have been previously developed or implemented for deconvolution: MuSiC [50], Robust Partial Correlations (RPC) [51], weighted robust linear regression (wRLM) [52], CIBERSORT [53], and non-negative least squares (nnls) [54]. For marker-based method, we considered debCAM-marker [12], TOAST-marker [55], and gsva [56]. For Bayesian method, we explored the recently published BayesPrism [15].

Procedures to evaluate deconvolution results vary in terms of whether the agreement between ground truth and inferred proportions is assessed by correlation or squared error and whether performance is evaluated per-cell type or globally. We focused our evaluation on per cell-type Pearson correlation, which reflects the accuracy of downstream inference such as the difference in proportions between two groups. We also calculated root mean square error (RMSE) values, which evaluate if the inferred proportions are correct on the absolute scale across different cell types, with smaller RMSE indicating better performance. The deconvolution pipelines including simulation, deconvolution, and evaluation are then applied to eight published single-cell cohorts (Table 1) and repeated 10 times for each cohort.

### Regression-based methods differ in their robustness to heterogeneity

Regression based approaches dominate the deconvolution field with many available methods and extensive independent benchmarking [9, 50, 57]. All regression methods fit a model that assumes that bulk expression matrix $Y_{g \times s}$ with *g* genes and *s* samples, can be expressed as a $Y_{g \times s}=X_{g \times k} P_{k \times s}+E_{g \times s}$, where *P* is the proportion matrix and *k* is the number of cell-types. *X* is the given reference matrix and the task is to fit *P*, which is a general regression problem.

A first decision in regression-based approaches involves the construction of the reference matrix *X*, a process termed as hyper-parameter reference construction. Selecting an optimal subset of genes is crucial for effective performance [58], while utilizing the entire gene set often results in poor outcomes (data not shown). The second methodological choice is the form of the regression problem itself, whether to use constraints, feature weights, and how to formulate the loss. For example: squared loss with constraints gives the basic non-negative least squares formulation (nnls). Alternative approaches involve weighted regression, robust regression, epsilon-insensitive loss (CIBERSORT), etc. Importantly, since the feature selection and regression are decoupled, we can combine different methods arbitrarily.

In our benchmarking work, we investigated four different reference matrix construction methods: CIBERSORTx [59], autogeneS [60], and two marker selection methods derived from differential expression (DE) analysis: limma [61] and scran (see the “Methods” section for details). Additionally, we considered an “all genes” setting, which is applicable exclusively to MuSiC, where all genes are utilized by default and no feature selection is performed.

**Fig. 3:**
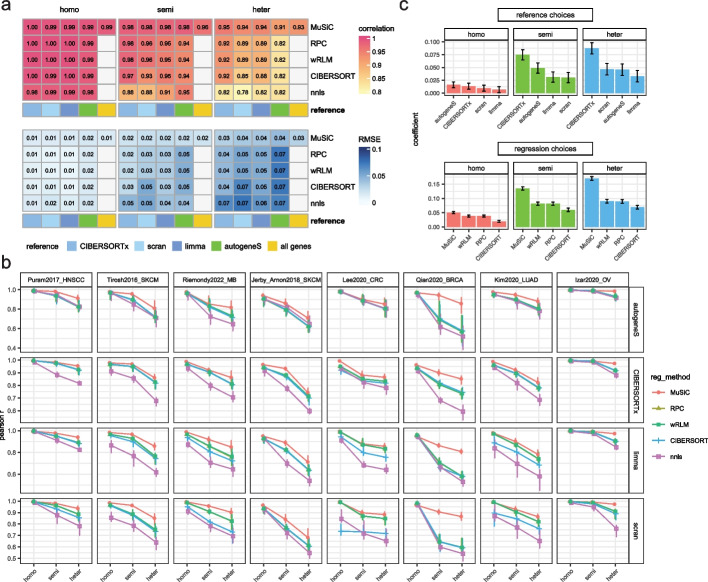
Impact of bulk simulation strategies on regression based deconvolution. **a** Heatmap comparing the deconvolution performance of regression models (rows) under different bulk simulation strategies, as evaluated by average Pearson correlation (upper panel) and average RMSE values (lower panel) over 10 experimental repeats for the Puram2017_HNSCC dataset. The columns of the heatmaps correspond to different reference construction methods, with the “all genes” column being specifically applicable to MuSiC, suggesting that all genes will be used as the input, which is the default setting for MuSiC. **b** Line plot comparing Pearson *r* of regression-based methods under various simulation strategies across eight different datasets, with each row representing a reference construction choice and the error bars indicating the min and max level of Pearson *r* over 10 experimental repeats. **c** Box plot showing the effect size (estimated by coefficients from the multivariate regression model) of different methodological choices in deconvolution performance across different simulated bulk conditions. The error bars indicate the 95% confidence intervals of the coefficients

Implementing these reference construction approaches to different regression methods, we systematically examined all possible combinations of these methodologies across eight distinct cohorts (Table 1), with 10 simulations for each cohorts (excluding “all genes” from methods other than MuSiC). A representative result using bulk data simulated from the Puram2017_HNSCC dataset is presented in Fig. 3a. We found that in the homogeneous setting, the results from different methodological combinations are remarkably consistent, with correlations spanning from 0.98 to 1. However, the performance of methods diverges with increasing heterogeneity. The results on all datasets is presented in Fig. 3b, where we showed that the performance of all methods decays as heterogeneity is added, while some methods decay less rapidly. We further summarized the influence of various methodological decisions by fitting a multi-linear regression to the Pearson correlation results with dataset and cell-type as a covariate (Fig. 3c; see the “Methods” section).

Altogether these results revealed that effect of methodological choices to be more pronounced with increased heterogeneity levels. Considering the summary in Fig. 3c, we find that in the homogeneous setting the reference choice method contributes relatively little (0.0067 to 0.016) and the coefficients are not significantly different. However, as heterogeneity levels in the bulk samples increase, the choice of reference becomes increasingly important and the CIBERSORTx reference dominates compared to other methods. We also note that the recently proposed optimization-based method autogeneS is indeed best in the homogeneous setting but is no better than differential expression-based method under heterogeneous settings.

Regarding regression choices, MuSiC consistently outperforms the others, irrespective of the chosen reference, under heterogeneous settings (Fig. 3b, c). On the other hand, nnls demonstrates the highest sensitivity to heterogeneity, exhibiting a sharp decline in performance as heterogeneity escalates and consistently ranking as the least effective among the regression models. The method of intermediate performance wRLM, RPC, and CIBERSORT are conceptually similar in that they make the regression problem robust in the technical statistical sense of robustness to outliers. This achieved by altering the loss function from squared loss to a function that grows less rapidly: epsilon insensitive loss for CIBERSORT and Huber loss for both RPC and wRLM (both use R based “rlm” function with default parameters). Overall these three conceptually similar approaches are indistinguishable when using sophisticated reference algorithms (CIBERSORTx or autogeneS, Fig. 3b top two rows), except that CIBERSORT regression lags behind the other two on DE-based references in four out of eight datasets. In Additional file 1: Fig. S11, we expanded our analysis to include another robust regression-based method, FARDEEP [62], which utilizes adaptive least trimmed squares in its optimization. Similar to the results discussed above, all robust regression-based methods demonstrated comparable sensitivity to changes in heterogeneity levels, each exhibiting decreased deconvolution capability in heterogeneous settings.

Notably, the MuSiC weighted regression which performs best differs conceptually from other regression approaches. Rather than altering the loss MuSiC weights the features based on the variance/covariance proprieties in the reference data. Our analysis showed that while the improvement afforded by this more complex approach is negligible in the homogeneous setting, its advantages are clearly evident in the heterogeneous one. Results were consistent across correlation and RMSE (Additional file 1: Fig. S10),

Finally, we note that the effects of the two regression methodological choices are additive. While MuSiC is originally designed to work with all genes—the only method capable of yielding satisfactory outcomes in an unfiltered context, adding an additional feature selection step further increases its performance. Specifically, the MuSiC (regression choice) and CIBERSORTx (reference construction choice) combination stood out as the best overall. We will refer to this combination as “MuSiC_CIBERSORTx” to highlight the difference from “MuSiC_default.”

### Marker-based methods are robust to heterogeneity

Marker-based methods represent a conceptually different class. Instead of solving a regression problem, these methods infer the cell type proportions based on the aggregate behavior of cell-type specific genes. The approaches can be broken down into two steps: selection of cell-type specific gene sets and the summarization of these gene sets. Depending on the summarization method the output may be either unitless scores (e.g., gsva [56]) or adhere to a sum-to-one constraint, providing a direct estimate of proportions that can be assessed using RMSE.

Since lack of proportion estimates is a major criticism of marker-based methods, we focused our analysis on two methods that report proportions: debCAM-marker (referred to as debCAM) [12] and TOAST-marker (referred to as TOAST) [55]. Additionally, we considered gsva [56], which is a widely used score-based method that does not provide proportions. For gene set selection process, we employed the same approaches that were applied in the reference construction for reference-based methods. Instead of taking quantitative expression values, in this step we only considered list of genes associated with each cell type: given a reference, we assigned each gene from the reference to the cell-type with the highest expression (see the “Methods” section).

**Fig. 4:**
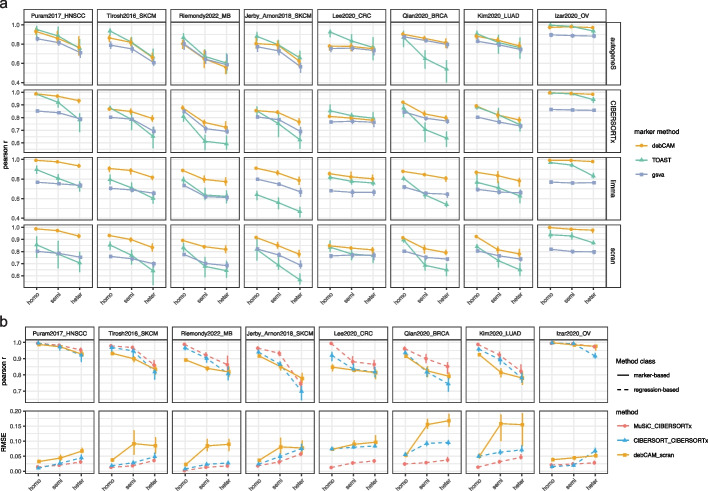
Impact of bulk simulation strategies on marker based deconvolution. **a** Line plot comparing Pearson *r* of marker-based methods under different simulation strategies across eight different datasets, with each row representing a marker construction method and the error bars indicating the min and max level of Pearson *r* over 10 experimental repeats. **b** Line plot comparing the best-in-class marker based method (debCAM_scran) with two regression based methods (MuSiC_CIBERSORTx and CIBERSORT_CIBERSORTx), with the performance being evaluated by Pearson *r* and the error bars indicating the min and max level of Pearson *r* over 10 experimental repeats

Overall, our findings revealed that performance varies significantly across different combinations of marker selection and summarization methods, with debCAM emerging as the best summarization technique (Fig. 4a, Additional file 1: Fig. S12). Specifically, the combination of debCAM and scran-based gene set selection consistently delivered the best overall results. Similar to regression-based approaches, we observed a decline in performance with the introduction of heterogeneity in simulations. However, unlike regression-based methods where performance disparities are only apparent under heterogeneous conditions, the relative performance of marker-based methods remains stable across both homogeneous and heterogeneous settings, with debCAM consistently excelling.

Finally, we compared best-in-class marker-based method (debCAM_scran) with two regression-based methods, MuSiC_CIBERSORTx, the best composite method we proposed and CIBERSORT_CIBERSORTx, the default CIBERSOTx method that has been widely used (Fig. 4b). Using Pearson correlation as the evaluation metric, our results indicated that while debCAM does not outperform regression-based methods in homogeneous simulation settings, it is highly competitive in heterogeneous settings, matching or even exceeding the performance of CIBERSORTx. This suggested that the gene set scoring technique employed by debCAM shows robustness against sample heterogeneity, even if it uses less information compared with regression-based methods. However, we also observed that debCAM demonstrates more variation in the RMSE values across experimental repeats, and generally exhibits higher RMSE compared to regression-based methods. This suggested that while debCAM accurately captures correlations, it is less effective at capturing the correct magnitude of the cell type fractions. Indeed, for debCAM, the absolute fraction inference is performed as a post-hoc adjustment to the scores; therefore, it does not consistently yield accurate estimations.

We note that the unitless marker-based method “gsva,” which typically exhibits the lowest performance when assessed through Pearson correlation, actually correlates non-linearly with the actual cell fractions. When evaluated using the Spearman correlation metric, the performance of “gsva” becomes considerably comparable to other marker-based methods, underscoring its potential for preliminary assessments of relative cell type proportions (Additional file 1: Fig. S13).

Additionally, we investigated the marker-based method “xCell” [10], which employs a built-in reference for estimating cell-type abundance. We compared the ground truth fractions with the matched cell-type signatures and observed that xCell signatures effectively predicts the true abundance within each immune cell type (Additional file 1: Fig. S14), Furthermore, most abundance estimates are highly coordinated between homogeneous and heterogeneous simulated samples, indicating its applicability as a preliminary investigation of bulk samples with unknown composition or unavailable single cell data. However, it is worth noting that xCell does not provide malignant fraction estimation and multiple xCell signatures can be mapped to the same cell type, making it difficult to distinguish and interpret the relevant signatures. For example, multiple B-cell related signatures are found to be closely correlated with B cell fractions (Additional file 1: Fig. S14).

Overall, we found that some marker-based methods are competitive with regression-based approaches and in some cases can offer advantages such as not requiring precise knowledge of reference values.

### Comprehensive assessment of deconvolution performance across conceptual classes

So far, we have performed a detailed evaluation of two major categories of deconvolution methods: the regression-based and marker-based approaches. In our final evaluation, we also included two additional classes: reference-free and BayesPrism, which uses a quantitative reference in a unique way that sets it apart from conventional regression-based approaches and thus constitutes its own class. While regression approaches fit an equation of the form $Y=XP+E$, where *E* represents error, BayesPrism solves a Latent Dirichlet Allocation (LDA) problem allocating all of the observed gene expression to a cell type so there is no residual [15]. Moreover, BayesPrism differs from conventional regression-based methods in that it uses all genes by default and its performance is not improved by subsetting (data not shown).

Since regression-based and marker-based methods are dependent on the feature selection procedure, for this evaluation we used the best performing choices. We selected reference matrices constructed with CIBERSORTx and markers generated from scran-derived DE analysis. For MuSiC, we included both the default implementation which uses all genes and the composite approach MuSiC_CIBERSORTx.

**Fig. 5:**
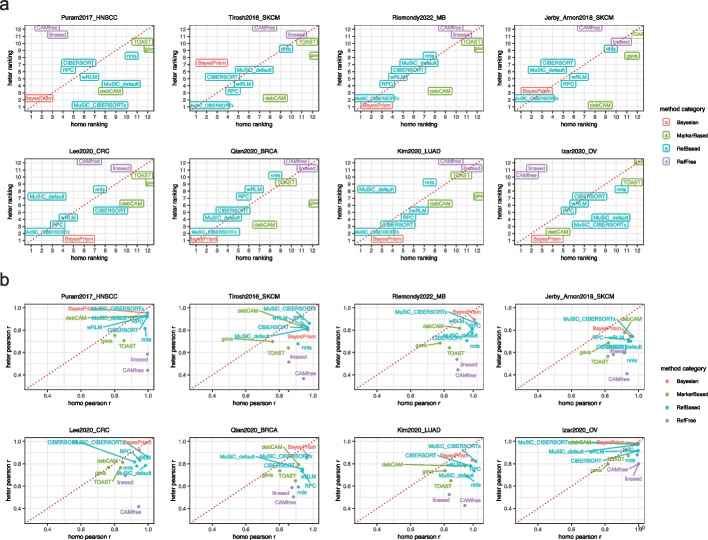
Deconvolution performance comparison under homogeneous and heterogeneous simulation. Scatter plot comparing **a** the ranking and **b** the average Pearson correlations of different deconvolution methods under homo and heter simulations, where the correlations are averaged over multiple experimental repeats. Different colors indicate different categories of deconvolution methods. All the regression-based methods are using CIBERSORTx-derived reference and all the marker-based methods are using scran-derived markers, while “MuSiC_default” means the default MuSiC setting where all the genes are being used as input

We summarized the final results both in terms of average Pearson correlations and relative rankings. The comparison between homogeneous and heterogeneous conditions is insightful for assessing shifts in performance under varying simulation scenarios and determining whether performance is influenced by changes in heterogeneity levels. Focusing on the rank-based comparison (Fig. 5a), methods that demonstrate consistent performance in both settings are located diagonally, while methods with significant performance disparities are positioned off-diagonal, for example, methods on the top left excel in homogeneous conditions but underperform in heterogeneous scenarios. Across dataset being tested, the relative rankings of different methods can be indeed drastically different under different simulation settings.

Specifically, the reference-free methods (purple) appear to perform well on some homogeneous datasets but they are ranked towards the bottom in the heterogeneous settings in *all cases*. This pattern suggested that their performance is highly context dependent and can be easily impacted by the increased heterogeneity level. Reference-free methods are conceptually attractive and continue to be developed and our simulation approach thus provides a valuable evaluation platform.

One the other hand, marker-based methods (green) are clearly overrepresented among those methods whose ranking improved in the heterogeneous setting. Maker-based methods are widely used [63] despite the advance of more sophisticated and more accurate approaches. Our analysis showed that they are particularly resilient to heterogeneity, which is likely an important property contributing to their popularity.

Out of all the methods being evaluated, we found that the rankings of BayesPrism and MuSiC (with CIBERSORTx reference) are consistently either unaffected or improved under heterogeneous settings.

We also provided the quantitative correlation plot as ranking can artificially inflate small differences. In the correlation based visualization (Fig. 5b), as expected we observed a systematic decrease in performance in the heterogeneous setting across all methods. It is not unusual to get correlations near 1 in the homogeneous setting, reflecting performance levels that can be unrealistically high, whereas in heterogeneous environments, correlations noticeably decline. This observation is further supported by a quantitative comparison of RMSE values under both homogeneous and heterogeneous settings (Additional file 1: Fig. S15). We expect that this indeed reflects real world performance. When deconvolution methods are tested against independent measurements of proportion as can be done with the malignant cell fractions in TCGA samples, even the best-performing methods BayesPrism does not achieve a correlation of $>0.8$ [64]. Thus, beyond ranking methods the heterogeneous simulation provides value in producing realistic performance estimates.

Overall, we found that BaysePrism and MuSiC_CIBERSORTx stand out as best overall. BayesPrism ranks first in the heterogeneous setting in 5 out of 8 datasets and is in the top 3 in another 2. MuSiC_CIBERSORTx is another top performer ranking in the top 2 for 5 datasets and always in the top 3. We note that in the ranking performance of BayesPrism on Tirosh2016_SKCM is ranked 7 and appears inconsistent with this assessment. However, from quantitative correlation plot the low performance of BayesPrism on Tirosh2016_SKCM is less remarkable as it is quantitatively similar to other top performing methods. A detailed performance summary for all methods under heterogeneous setting can be found in Additional file 1: Fig. S16.

Taken together, our findings emphasized the impact of heterogeneity on deconvolution results and indicated that benchmarking using homogeneous samples may not accurately represent real-world performance. In realistic bulk simulation scenarios, the Bayesian method BayesPrism and the regression methods MuSiC stand out as the top-performing approaches. These results shed light on the importance of considering heterogeneity when evaluating and selecting deconvolution methods for practical applications.

## Discussion

In this study, we introduced the importance of heterogeneity in bulk sample simulation and examined how heterogeneity could influence the deconvolution results. We investigated four major categories of deconvolution methods by applying them to simulated samples with different heterogeneity levels and identified the top performing ones. Our results showed that introducing biological heterogeneity has a notable effect on deconvolution performance, leading to a global performance drop as heterogeneity level increases, while some methods being more robust to this change.

Specifically, BayesPrism is one of the top-performed method across all the datasets we tested. Our results align with a recent study [65] that benchmarked deconvolution methods on real bulk and single cell data finding that BayesPrism strongly outperforms all tested reference based methods when evaluated for consistency across different biochemical and bioinformatic processing pipelines for the same biological sample. We reasoned that this can be attributed to its explicit consideration of heterogeneity within the malignant cells. The novelty of incorporating “cell-state” information within the same cell-type and reporting the posterior sum over various cell states makes BayesPrism particularly effective in handling heterogeneous settings.

Another method that exhibits high robustness in our evaluation is MuSiC, which also accounts for heterogeneity within a cell type. Specifically, MuSiC employs a weighting scheme that prioritizes genes with low cross-subject variance. It then solves the deconvolution task as a weighted non-negative least squares regression problem. Although it has been proposed that pre-selecting marker genes for the weighted regression is not necessary, our findings indicated that incorporating marker genes pre-filtered from CIBERSORTx can significantly enhance the deconvolution performance of MuSiC.

In addition to the aforementioned methods, it is worth noting the outstanding performance of the marker-based method debCAM. It ranked in the top 3 in 6 out 8 datasets surpassing many reference-based methods. It may appear counter-intuitive that maker-based methods can outperform regression-based ones as marker-based methods seemingly use less of the available prior information. However, in the heterogeneous setting, this may be an advantage. In the heterogeneous setting, the regression objective becomes only an approximation as the true cell-type means are altered and genes have considerable residual covariance. The various feature selection and weighing schemes using by top performing methods serve to account for this but may do so imperfectly. On the other hand, marker-based methods are highly robust to these effects as the residual covariance of marker genes is low by construction and the exact mean values are not relevant.

In line with the view, BayesPrism presents an interesting case of a method that is fully quantitative but has some conceptual similarity with marker-based approach. BayesPrism uses the full reference matrix but does so in a way that does not rely on the exact reference values. One of the sampling steps of BayesPrism involves distributing the counts in the observed bulk expression for a gene over the current estimate of cell-type specific contributions with a multinomial distribution [66]. As such the absolute scale of gene expression in the reference matrix is not relevant, as the values are interpreted as probabilities and normalized to sum to 1. Moreover, in this setting, the contribution of a single gene to the final proportion estimate is directly proportional to its relative cell-type specificity times its expression value in the bulk sample. Consequently, the BayesPrism approach to a large degree negates the model misspecification sensitivity of regression-based methods.

Finally, our finding suggested that reference-free methods perform poorly under the heterogeneous scenario. Reference-free methods are attractive as they require no prior knowledge and it has been repeatedly suggested that these methods produce reliable proportion estimates [13, 14]. However, we showed that the more realistic the simulation strategy the accuracy is much lower than would be expected from the previously reported results. Reference-free methods rely on fitting a simplex structure which is the expected data geometry if the only source of variation is cell-type proportions. However, adding variation beyond cell-type proportions introduces additional lower dimensional structure making the proportion associated simplex difficult to isolate.

Our analysis provides valuable insights into the performance and tradeoffs of different conceptual approaches in a highly realistic simulation scenario thus establishing a framework for future methodological development. Beyond the specific deconvolution problem addressed in this work, the heterogeneous simulation strategy can be employed in other simulation pipelines to produce more realistic performance benchmarks for additional tasks such as cell-type specific differential expression [67].

We also acknowledge some limitations of our approach. While we demonstrated that our heterogeneous simulation strategy matches the variance observed in real bulk samples, not all aspects of real data will be preserved. For example, the dependencies between cell types can be violated as we randomly combine cell types from different patients. Chu et al. [15] found that certain biological pathway activation in malignant cells could be negatively correlated with cell type fractions of other non-malignant cells and the heterogeneous simulation we propose does not take into account such correlations. Methods that overcome this limitation would need to take the ground truth cell-type covariance into account necessitating development of new proportion sampling strategies.

Additionally, our study did not directly account for potential batch effects between the single-cell data used to generate references and the bulk data undergoing deconvolution. The benchmarking framework we introduced was based on a simplified scenario where matched single-cell data is available for reference construction. In real practice, there could be technical variation between signature matrices and bulk mixtures due to differences in assay platform [59, 68]. A complete evaluation for complex prior mis-specification will be the subject of future work.

## Conclusions

Overall, our work suggests specific recommendations for creating realistic bulk simulations and highlights counterintuitive findings regarding the performance of deconvolution approaches from different conceptual classes. Together, we expect that these contributions will provide the groundwork for future methodological improvements.

## Methods

### Single-cell RNA seq datasets and quality control

A total of eight single-cell RNA sequencing datasets from seven tumor types are used in this paper (Table 1): (i) head and neck squamous cell carcinomas (HNSCC) from Puram et al. [25], (ii) melanoma (SKCM) from Tirosh et al. [29] and Jerby-Arnon et al. [33], (iii) medulloblastoma (MB) from Riemondy et al. [31], (iv) colorectal cancer from Lee et al. [35], (v) breast cancer from Qian et al. [37], (vi) lung adenocarcinoma from Kim et al. [39], and (vii) ovarian cancer from Izar et al. [41]. A detailed description of single-cell datasets used in this paper can be found in Additional file 2: Table S1.

UMI counts were converted to counts per million (CPM) prior to downstream simulation tasks. We removed genes that are expressed in less than 5 cells and discarded genes from mitochondrial or ribosomal content. All expression matrices are in linear (non-log) scale.

For melanoma dataset Tirosh2016_SKCM, we used cell-type labels re-classified in Schelker et al.’s [69, 70] study. For MB dataset Riemondy2022_MB, we re-annotated the immune population based on immune cell subtyping information from the interactive website of the original paper [71]. We included major immune cell types from their annotations for further study: DC, Neutrophil, NK cell, T cells. For all the macrophage subpopulations: chemokine myeloid, complement myeloid, M2-activated myeloid, and non-activated microglia, we relabeled them into macrophages to ensure a reasonable resolution of cell types. Immune cells that are classified as “Proliferate” or do not have any subtyping label are excluded from further study. For ovarian cancer dataset Izar2020_OV, we excluded cell type “Erythrocyte” due to limited single-cell size. For the remaining scRNA datasets, we used their original cell-type labels.

### Bulk data used for variance comparison

When comparing the variance of the simulated bulk expression, we considered two types of “real” bulk data for comparison. First, baseline expression: we aggregated single cells from the same biological samples within the scRNA-seq data, which serves as an approximation of actual bulk samples [72, 73]. Specifically, this was achieved by calculating the mean expression from the CPM-normalized expression of cells from the same biological samples. The resulting profiles maintain consistent library sizes and are ready for variance analysis. Second, real bulk expression data from The Cancer Genome Atlas (TCGA): we accessed the TCGA cohorts from https://xenabrowser.net/. The gene expression data was acquired in HTSeq-FPKM format and subsequently transformed to Transcript Per Million (TPM) for variance comparison. Only samples from primary and metastatic sites were selected for the analysis. A detailed description of the datasets used for variance comparison can be found in Additional file 2: Table S2.

### Simulation of cell-type frequencies

#### Cell-type fractions simulation from beta distribution

To introduce variances into the cellular compositions of simulated bulk samples, we simulated cell-type frequencies that are close to that in real bulk samples. The cell-type proportions of each patient from the scRNA dataset were used as an approximation to the cell-type frequencies of real bulk samples. We fitted a beta distribution for each cell type and drew random values from the fitted distribution as the simulated frequencies. Randomly selected frequencies for different cell types are then scaled and summed to one for each simulated bulk sample. This fraction simulation method, refereed to as “beta” method in Additional file 1: Fig. S3, is implemented throughout the simulated bulk expression in this study.

#### Cell-type fractions simulation from external resources

In Additional file 1: Fig. S3, we evaluated the performance of our fraction simulation approach by comparing it with an external method employed by Avila Cobos et al. [9] (referred to as “favilaco”), and a basic fraction simulation utilizing the Dirichlet distribution. The “favilaco” method, which is part of the bulk simulation pipeline proposed by Avila et al., is now available as the $bulkSimulator\_favilaco()$ function in the deconvBenchmarking package. This function simultaneously simulates bulk expression data and fractions, which we used to extract and compare the simulated fraction distributions with other methods.

For the Dirichlet-based simulation, we set the shape parameter $\alpha$ to reflect the relative abundance of each cell type. Specifically, $\alpha$ is set to the cell type frequencies from the single-cell dataset used for bulk simulation and adjusted by a dispersion parameter to modulate the spread of the distribution. We considered a set of varying levels of dispersion parameters 0.01, 0.05, 0.001 in the simulation. The Dirichlet-based simulation was conducted using the $fracSimulator\_Dirichlet()$ function from the deconvBenchmarking package.

By comparing the distribution of the simulated fractions under different settings, we showed that “favilaco”-based method significantly deviates from the baseline’s mean and variance, and while Dirichlet distribution exhibits comparable distributions, it requires additional tuning of the dispersion parameter, making it less efficient than the beta distribution based method we proposed.

### Bulk simulation strategies

Using the same source scRNA-seq dataset and simulated cell-type fractions, each simulated bulk expression in this study was comprised of 100 simulated samples, created using the following strategies:

#### Homogeneous simulation

We standardized the widely used bulk simulation method that aggregates over random cells as homogeneous simulation. Specifically, in each simulated sample, *n* single cells are aggregated linearly, with their proportions aligned to the simulated cell-type frequencies. The value of *n* is set to approximate the typical number of single cells of a biological sample from the scRNA data (Additional file 2: Table S1).

#### Semi-heterogeneous simulation

We restricted that the malignant parts of each simulated bulk sample come from the same patient, while the non-malignant parts are randomly selected regardless of where they are from. Specifically, for each simulated bulk sample *i*, the malignant expression signal come exclusively from a randomly selected patient’s malignant profile $C_{malignant}$ and is weighted according to the simulated malignant fraction, and the non-malignant single cells are randomly selected and weighted according to the corresponding simulated frequencies.

#### Heterogeneous simulation

We restricted that both malignant and non-malignant parts of each simulated bulk sample come from the same biological sample. Specifically, for each simulated bulk sample *i*, given a cell-type *k*, the expression signal of cell-type *k* comes exclusively from a randomly selected patient’s *k* profile $C_k$ and is weighted according to the simulated fraction (Additional file 1: Fig. S2).

Note that in both semi-heterogeneous and heterogeneous simulation settings, we employed additional randomization to prevent using the exact same cells across different simulated samples. Specifically, when aggregating patient-specific single cells to create a unique patient profile, we randomly select between 50 and 100% of the single cells. Moreover, we introduced a threshold parameter to specify the limited number of cells used for aggregation. If the number of patient-specific single cells falls below this threshold, we aggregate across multiple samples to prevent sparsity issues [44] within the cell-type specific profile. These two methods are now implemented as the $bulkSimulator\_semi()$ and $bulkSimulator\_heter()$ function in the deconvBenchmarking package.

#### sampleID independent heterogeneous simulation

We restricted that each cell-type component in the simulated bulk sample is constrained to originating from the same sub-cluster. Specifically, sub-cluster information for each cell type is obtained using the *quickCluster*() function from scran R package, with the *min*.*size* parameter set to 10 [74].

#### Bulk simulation from external resources

We incorporated three additional published approaches for bulk simulation comparison: the “favilaco” method [9], the “immunedeconv” method [17], and the “SCDC” method [23]. Note that only the “immunedeconv” method supports user-provided fractions; therefore, we passed the simulated fractions to this method; the “favilaco” and “SCDC” methods do not support user-provided fractions, so we retained their default settings in the bulk simulation. These methods are implemented as $bulkSimulator\_favilaco()$, $bulkSimulator\_SCDC()$, and $bulkSimulator\_immunedeconv()$ function in the deconvBenchmarking package, which we used for bulk simulation.

### Calculation of biological variance in bulk samples

The following statistics are calculated as indicators of biological variance. Note that the baseline expression referred to below represents pseudobulk samples from single-cell expression, which is obtained by aggregating single cells from the same patients, as an approximation of real bulk sample.

#### Coefficient of variation (CV)

For each gene *i* in the simulated and baseline bulk samples, we calculated CV values on the log transformed expression using the following formula:

$$\begin{aligned} CV_{i} = \frac{\sigma _i}{\mu _i} \end{aligned}$$

#### Average coefficient of variation (CV) for biological pathways

We downloaded the hallmark gene list from https://www.gsea-msigdb.org/gsea/msigdb/ and calculated the average CV values for genes included in each genelist, which is used as indicators for pathway-level variance.

#### Pairwise correlations between genes

We considered two sets of genes for calculating the gene-gene correlation matrix: (1) the top 300 most variable genes from the baseline bulk expression and (2) the cell type marker genes derived from limma-based differential expression analysis. The detailed listing of the genes used can be found in Additional file 2: Table S3. Within each simulated bulk expression and the baseline expression, we calculated the Pearson correlations between these genes and visualized them in a heatmap [75, 76].

#### Pairwise similarities between samples

Using the top 300 most variable genes from the baseline bulk expression, we calculated the pairwise Pearson correlation between samples for each simulated bulk expression. We then visualized the distribution of these statistics using a boxplot (Additional file 1: Fig. S7).

### Reference construction

To generate necessary input for reference-based methods (regression-based, marker-based, and BayesPrism), we applied the following reference construction methods using the training cells.

In particular, for interchangeability between the signature matrices and cell type markers, we applied the $refMarkers\_sigMatrixList()$ and $refMatrix\_markerList()$ functions we developed in the deconvBenchmarking R package. Specifically, to create a signature matrix from a set of gene lists, we first averaged the expression values of single cells from the same cell type, resulting in a raw gene-by-cell-type matrix; we then refined this matrix by subsetting it with the marker genes. To identify cell-type markers from a given reference matrix, we assigned each gene to the cell type with the highest expression.

We note that for MuSiC, the regression-based method in our benchmarking, the values generated in the reference matrix are not directly used by MuSiC itself. Instead, they serve as a feature selection step, where the genes identified in the reference matrix are used as the *marker* input for MuSiC.

#### CIBERSORTx signature matrix

We used the “Create Signature Matrix” module from the online CIBERSORTx portal (https://CIBERSORTx.stanford.edu) to generate the signature matrix, with the training expression as input and all the parameters set to default values. We note that CIBERSORTx has a size limit for the input, so we downsampled the training cells and shrunk the input size when necessary. The resulting signature matrices are used directly as input for regression-based methods. Note that the CIBERSORTx-based reference typically contains thousands of genes in its signature. When converting this reference matrix to cell-type marker genes using the $refMatrix\_markerList()$ function, we employed the $maximum\_n$ = 100 parameter to ensure that each cell type can have at most 100 marker genes, prioritized by the fold change in the reference matrix.

#### autogeneS signature matrix

The python package autogeneS [60] is utilized for signature matrix construction. We used the following parameters in the *optimize* function: $ngen=3000, seed=0, mode = fixed, nfeatures=400$. The resulting optimized reference matrix with pareto index 0 is selected as autogeneS signature matrix.

#### limma derived cell-type specific markers

R package limma [61] is used to identify cell-type-specific markers that are differentially expressed within each cell-type. Specifically, the one-against-rest comparison is performed for the statistical test, comparing each cell-type against all other cell-types combined. Genes with a log fold change greater than 2 are considered as cell-type specific markers, and a parameter $minimum\_n = 5$ is introduced to ensure a sufficient number of markers for each cell type. Cell types with fewer than $minimum\_n$ genes passing the log2 fold change threshold will be excluded from the marker list. Note that for the Riemondy2022_MB and Jerby_Arnon2018_SKCM dataset, the log fold change threshold is relaxed to 1 to ensure proper number of cell-type specific markers.

#### scran-derived cell-type-specific markers

The *BayesPrism* :: *get*.*exp*.*stat*() function, which incorporates the *pairwiseTTests*() function from the scran package, is used to identify cell-type specific markers. Specifically, pairwise comparison between cell types is performed for the statistical test [74]. The marker gene filtering process here utilizes the same log fold change threshold and $minimum\_n$ parameter as applied in limma derived marker identification.

#### Reference for BayesPrism

We designated cell type labels from the single-cell dataset as *cell*.*type*.*label* and subclassified malignant populations based on their biological origins as *cell*.*state*.*label* in the *BayesPrism* :: *new*.*prism*() function, the same BayesPrism reference construction strategy used in Hippen et al.’s study [65]. We adhered to the reference derived directly from the scRNA data, bypassing the reference update step in BayesPrism, which entails generating a new reference from initial deconvolution results. This decision was based on our observation that utilizing the updated reference contributes minimally to performance enhancement (Additional file 1: Fig. S17).

### Highly variable (hv) genes

For computational efficiency, we selected highly variable genes as candidates to run autogeneS and DE analysis. We used the *plot*.*scRNA*.*outlier*() function from R package BayesPrism to calculate the maximum cell-type specificity score for each gene. Genes with *max*.*spec* greater than a threshold value (0.5 for autogeneS and 0.3 for DE analysis) are selected for downstream analysis. This filtering narrows down the gene candidates from more than 10 thousands to thousands.

### Deconvolution methods

Deconvolution methods are applied to the simulated bulk samples in their linear scale (non-log transformed) following recommendations from previous benchmarking studies [9, 16]. All methods evaluated in the study are wrapped in the deconvBenchmarking R package we developed: https://github.com/humengying0907/deconvBenchmarking.

### Evaluation of deconvolution performance

Pearson correlation and root mean square error (RMES) values are used to evaluate the accuracy of different deconvolution methods. Specifically, for Pearson correlation, we calculated per cell-type correlations by comparing estimated fractions to known fractions within each cell type, with higher Pearson *r* corresponding to better performance. This process results in a set of per cell-type correlations for each simulated bulk dataset. These correlation values are then averaged to yield the overall correlation performance score for a given deconvolution method. For RMSE values, we focus on a global comparison between the estimated and known fractions for all cell types altogether, with smaller RMSE values indicating a lower absolute difference and thus better performance.

The averaged Pearson *r* across experimental repeats is used to represent the overall performance of a method. Variation in Pearson *r* and RMSE values across experimental repeats is visually inspected by comparing the minimum and maximum level across 10 experimental repeats, which help evaluate the reproducibility and stability of each method.

For reference-free deconvolution methods, where cell-type labels are not explicitly provided, we calculated pairwise Pearson correlations between the estimated and the known fractions and assign the unnamed cell type to the cell type with which it showed the highest correlation (Additional file 1: Fig. S18).

### Multi-linear regression model of deconvolution performance

In order to evaluate the impact of methodological choices on deconvolution performance for regression-based methods, we utilized a linear modeling approach. Specifically, we fitted a linear model using the *lm*() function in R to predict Pearson correlation coefficients based on various predictors, incorporating dataset and cell types as covariates. The model formula employed was:

$$\begin{aligned} \text {model} = lm( \text {correlation} \sim 1 + \text {regression}\_\text {choice} + \text {reference}\_\text {choice} + \text {dataset} \times \text {cell}\_\text {type}) \end{aligned}$$

We designated nnls as the baseline regression choice, and no selection with MuSiC regression as the baseline reference choice for comparison. The coefficients obtained from the model indicate, conditional on all other effects, the relative impact of a specific methodological choice over the baseline. Higher coefficients indicate a more significant impact of this methodological choice on overall performance. The model was fitted separately for bulk data simulated from different approaches, in order to explore the effect shift of methodological choices under different conditions.

### Supplementary information

Additional file 1. Supplementary figures S1-S18 referenced in the main text.

Additional file 2. Supplementary tables S1-S4 referenced in the main text.

Additional file 3. Review history.

## Data Availability

All source code, simulated datasets, and detailed benchmarking results are available in Zenodo with DOI: 10.5281/zenodo.11238300 [77], under the open-source GPL-3.0 license. The deconvBenchmarking R package developed in this study is deposited at the GitHub repository: https://github.com/humengying0907/deconvBenchmarking [78], under the open-source GPL-3.0 license. All data used in this study were obtained from publicly available repositories: 1   Single cell head and neck cancer dataset Puram2017_HNSCC is downloaded from the GEO database under the accession code GSE103322 [28]. 2   Single cell melanoma dataset Tirosh2016_SKCM is downloaded from the GEO database under the accession code GSE72056 [30]; the single cell annotation data is downloaded from https://github.com/icbi-lab/immune_deconvolution_benchmark [69, 70]. 3   Single cell medulloblastoma dataset Riemondy2022_MB is downloaded from GEO database under the accession code GSE155446 [32]; the immune cells meta data is downloaded from the online interactive browser [71]. 4   Single cell melanoma dataset Jerby_Arnon2018_SKCM is downloaded from GEO database under the accession code GSE115978 [34]. 5   Single cell colorectal cancer dataset Lee2020_CRC dataset is downloaded from the 3CA database (https://www.weizmann.ac.il/sites/3CA/colorectal) under the Title identifier “Lee et al. 2020” [36]. 6   Single cell breast cancer dataset Qian2020_BRCA is downloaded from the 3CA database (https://www.weizmann.ac.il/sites/3CA/breast) under the Title identifier “Qian et al. 2020” [38]. 7   Single cell lung cancer dataset is downloaded from the 3CA database (https://www.weizmann.ac.il/sites/3CA/lung) under the Title identifier “Kim et al. 2020” [40]. 8   Single cell ovarian cancer dataset is downloaded from the 3CA database (https://www.weizmann.ac.il/sites/3CA/ovarian) under the Title identifier “Izar et al. 2020” [42]. 9   TCGA Melanoma cohort is downloaded from UCSC Xena Browser under the cohort identifier "GDC TCGA Melanoma (SKCM)" [79]. 10   TCGA Head and Neck Cancer cohort is downloaded from UCSC Xena Browser under the cohort identifier "GDC TCGA Head and Neck Cancer (HNSC)" [80]. 11   Hallmark gene sets used in this paper are downloaded from The Molecular Signatures Database (MSigDB): https://www.gsea-msigdb.org/gsea/msigdb/ [81].
