# Supplementary material for: Heterogeneous pseudobulk simulation enables realistic benchmarking of cell-type deconvolution methods
Source: Genome Biol. 2024 Jul 1;25:169. doi: 10.1186/s13059-024-03292-w (PMC11218230; doi:10.1186/s13059-024-03292-w)
Supplement: Supplementary file 1 — Additional file 1. Supplementary figures S1-S18 referenced in the main text. [file 13059_2024_3292_MOESM1_ESM.pdf]

Additional file 1: Supplementary Figures

Fig S1. Schematic framework of the benchmarking study

Fig S2. Schematic representation of heterogeneous bulk simulation

Fig S3. Comparison of fraction simulation strategies

Fig S4. Comparison of different bulk simulation strategies: Heatmap visualization of pairwise gene correlations

Fig S5. Comparison of different bulk simulation strategies: Variance in biological pathways

Fig S6. Comparison of different bulk simulation strategies: Scatter plot of variance comparison

Fig S7. Comparison of different bulk simulation strategies: Distribution of sample-wise correlations

Fig S8. Heterogeneity in malignant cells

Fig S9. Heterogeneity in non-malignant cells

Fig S10. Overall RMSE for regression-based methods under various simulation settings

Fig S11. Robust regression methods show similar sensitivity to changes in heterogeneity levels

Fig S12. Overall RMSE for marker-based methods under various simulation settings

Fig S13. Gsva score estimates correlate non-linearly with ground truth fraction

Fig S14. Pearson correlation of xCell signatures with ground truth fraction

Fig S15. Overall RMSE comparison under homogeneous and heterogeneous conditions

Fig S16. Detailed performance comparison under heterogeneous simulation

Fig S17. BayesPrism using initial vs updated reference

Fig S18. Pairwise correlation between fraction estimates and ground truth fractions facilitates cell-type mapping in reference-free methods

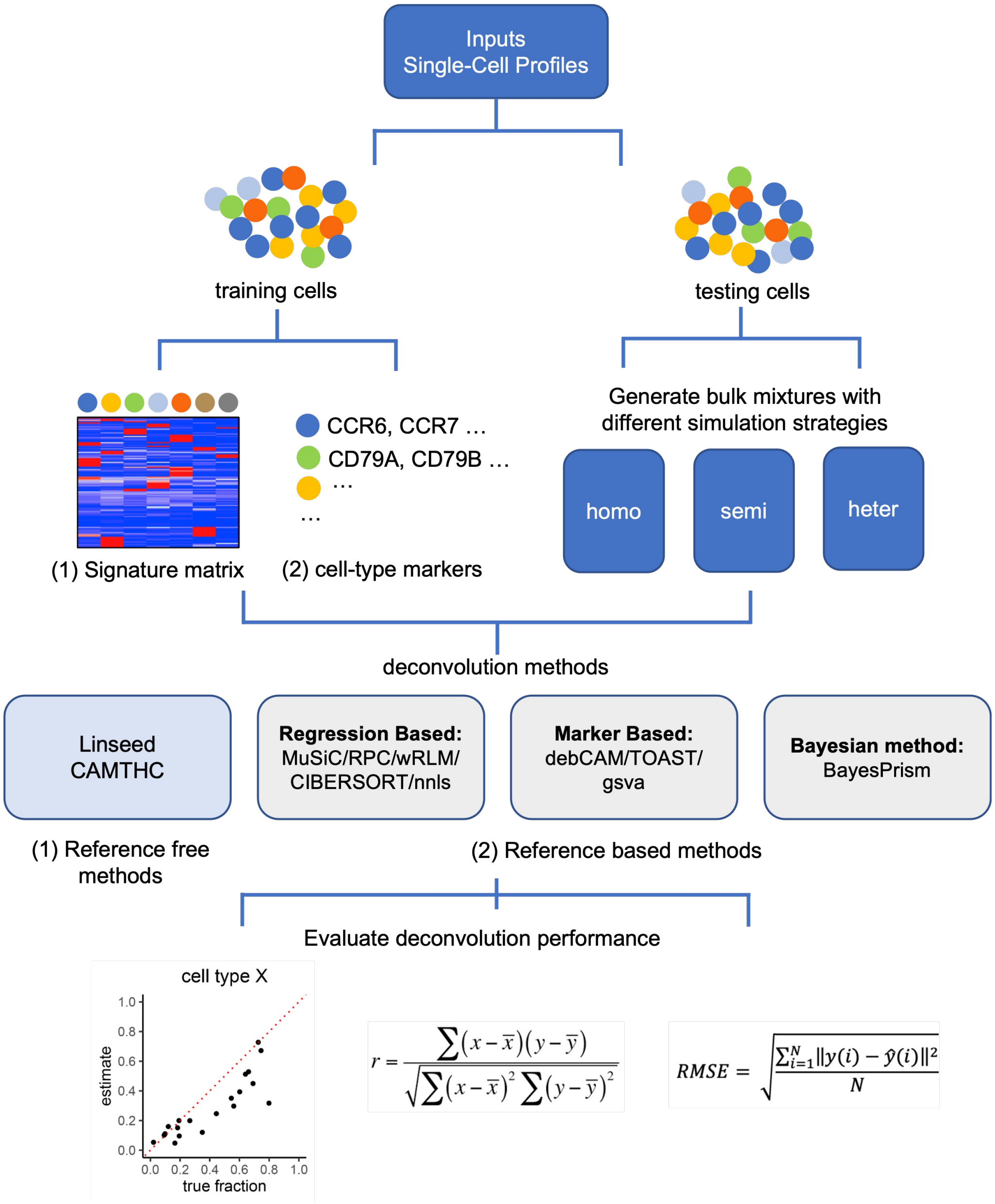

Fig S1. Schematic framework of the benchmarking study

The complete framework involves the following steps: **(1)** The benchmarking framework takes the single-cell expression matrix and predefined cell-type labels as input. **(2)** Single cell profiles are then split into training (50%) and testing (50%) sets. **(3)** Training set is used to create reference signature matrices consisting of informative marker gene expression, and cell-type marker list that can discriminate between different cell-types. Testing set is used to build simulated bulk samples using three different simulation strategies: homogeneous (homo), semi-heterogeneous (semi) and heterogeneous (heter) simulation. **(4)** Four categories of deconvolution methods are implemented: reference-free methods, regression-based methods, marker-based methods and Bayesian method. **(5)** Performance of different deconvolution methods is evaluated by comparing between the estimated and known fraction using the following statistics: Pearson correlation coefficient, root mean square error (RMSE).

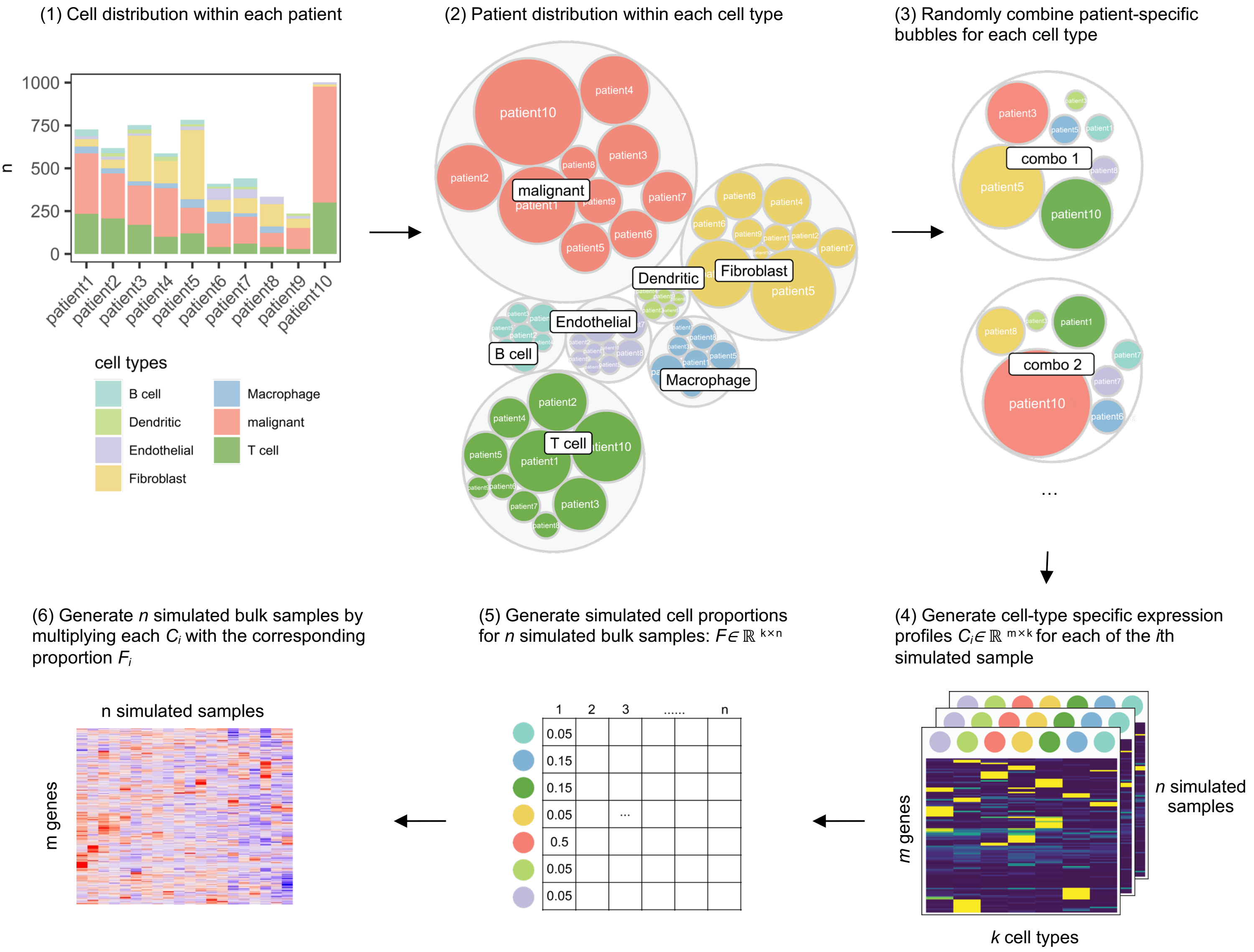

Fig S2. Schematic representation of heterogeneous bulk simulation

Heterogeneous bulk simulation involves the following steps: **(1)** cell-type distribution is first summarized at per patient level; **(2)** Patient barcode information is summarized at per cell-type level; **(3)** For each of the *i*th simulated sample, out of *n* total samples, single cells are randomly selected and aggregated. This selection is constrained such that each cell type comes from the same biological sample. For example, in the first simulated sample ("combo 1"), fibroblasts are randomly selected from Patient 5, malignant cells from Patient 3, etc. All single cells selected for "combo 1" are then used to construct this simulated sample; **(4)** Using the aggregated single cells from Step 3, we create cell-type specific expression profiles C_i for each combination of cells (referred to as "combo"). Specifically, for each “combo”, we take the average expression values of single cells from the same cell type to get the cell-type specific expression profiles $C_i \in \mathbb{R}^{m\times k}$ for the *i*th simulated sample. This process results in *n* individual cell-type specific expression profiles C_i; **(5)** Simulate cell-type proportions; **(6)** Each cell-type specific expression profile C_i is weighted by cell-type proportions *F* to yield the final heterogeneously simulated bulk expression.

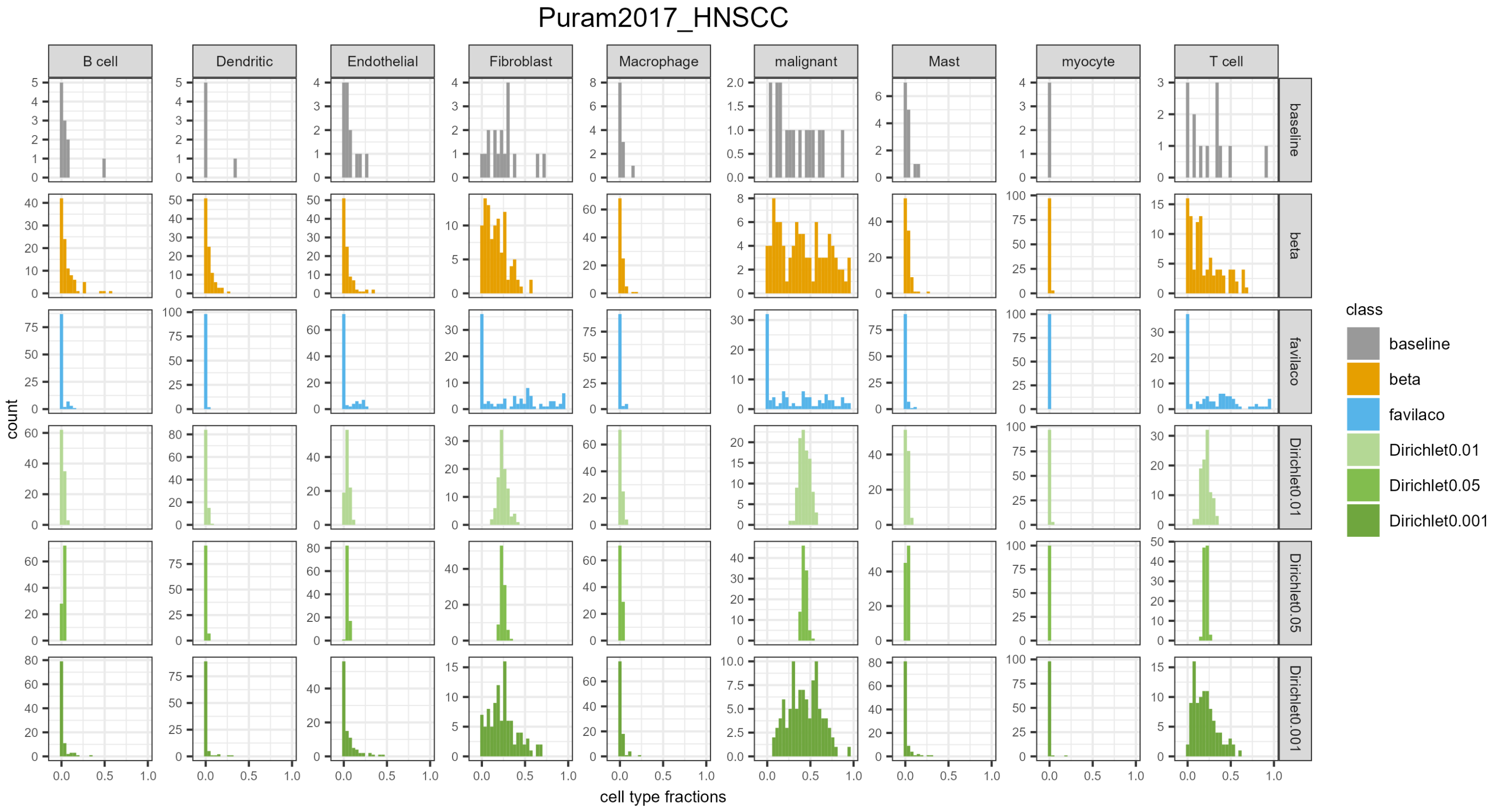

Fig S3. Comparison of fraction simulation strategies

Histogram comparing the distribution of cell-type proportions from an example dataset. The first row shows the cell-type proportions of each patient from the scRNA dataset, serving as a baseline for fraction comparison. The second row corresponds to beta-distribution based fraction simulation. This fraction simulation method is applied to bulk simulation throughout this study. Additional rows showcase the distribution of cell-type fractions obtained from alternative methods: the approach adopted by Avila Cobos et al. (labeled as "favilaco"), and the simulation from a Dirichlet distribution with varying levels of dispersion parameters.

a

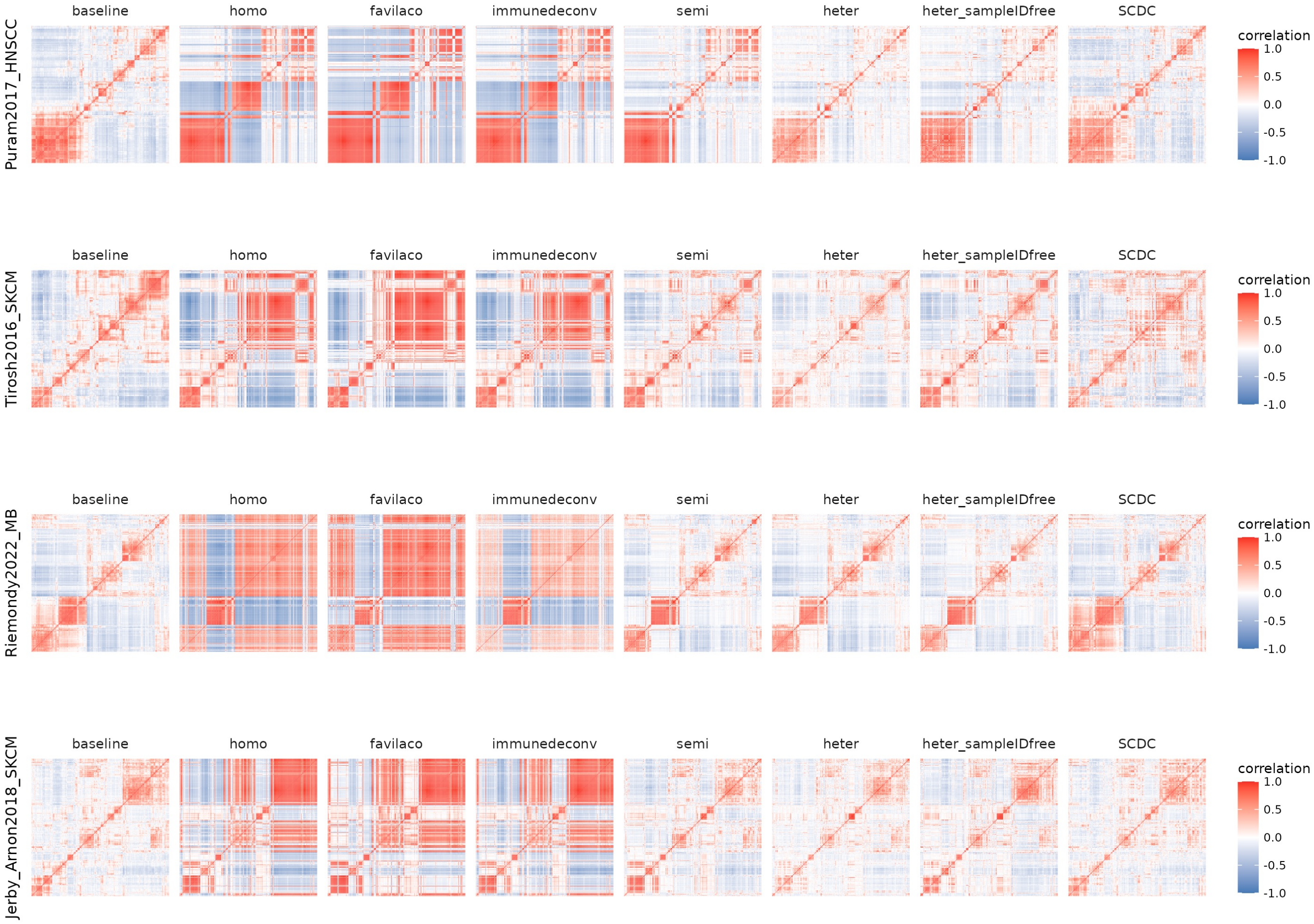
Puram2017_HNSCC
Tirosh2016_SKCM
Riemondy2022_MB
Jerby_Arnon2018_SKCM
baseline
homo
favilaco
immunedeconv
semi
heter
heter_sampleIDfree
SCDC
correlation
1.0
0.5
0.0
-0.5
-1.0

b

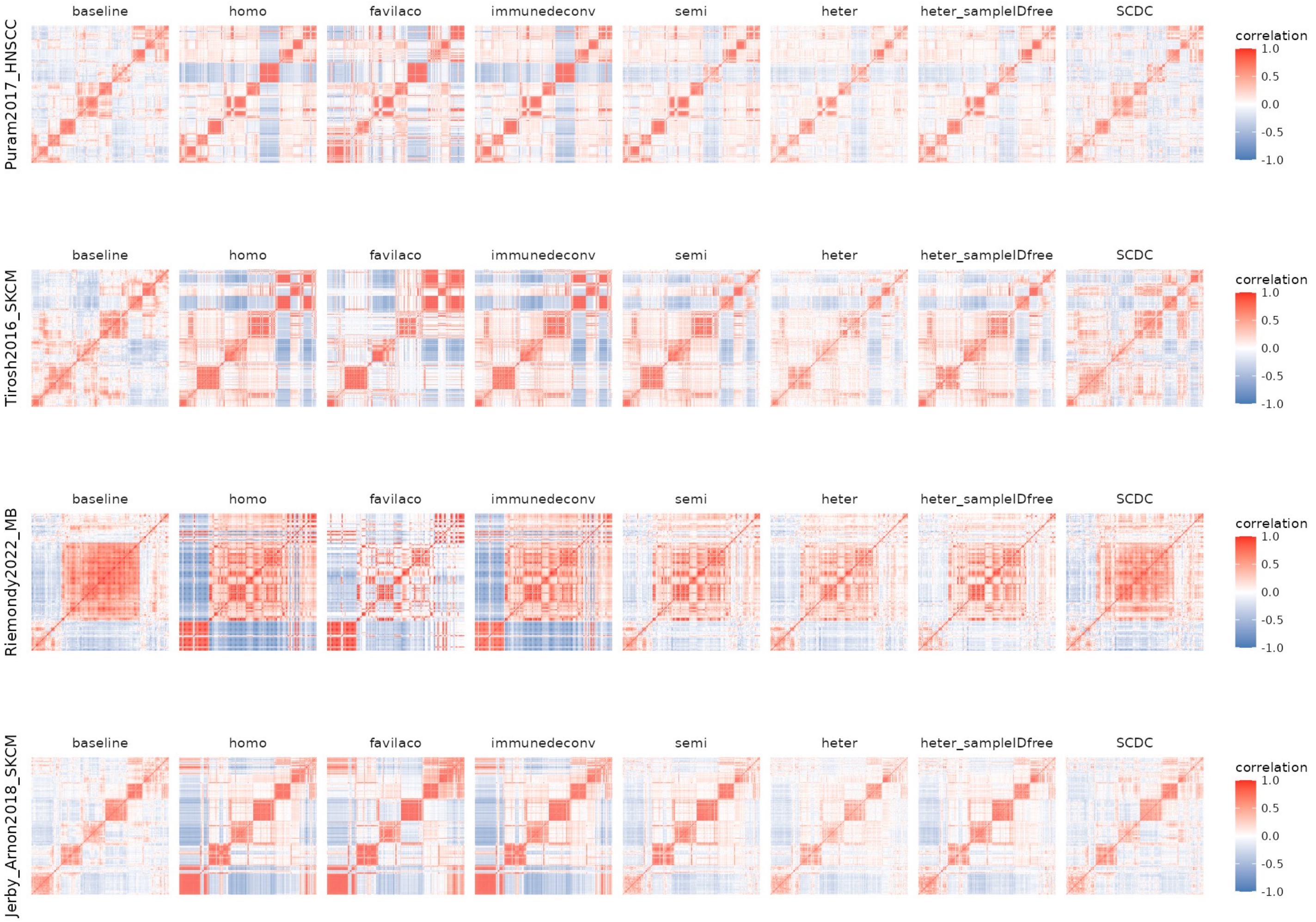

Fig S4. Comparison of different bulk simulation strategies: Heatmap visualization of pairwise gene correlations Heatmap showing gene correlations in baseline and simulated bulk expression across datasets. The baseline bulk expression is obtained from aggregated cells of the same patient in the single-cell cohort, as an approximation of real bulk expression. Each simulated bulk expression profile consists of 100 simulated samples. The **(a)** top 300 most variable genes from the baseline expression and **(b)** cell-type marker genes (derived from limma-based DE analysis) are selected for gene correlation comparison.

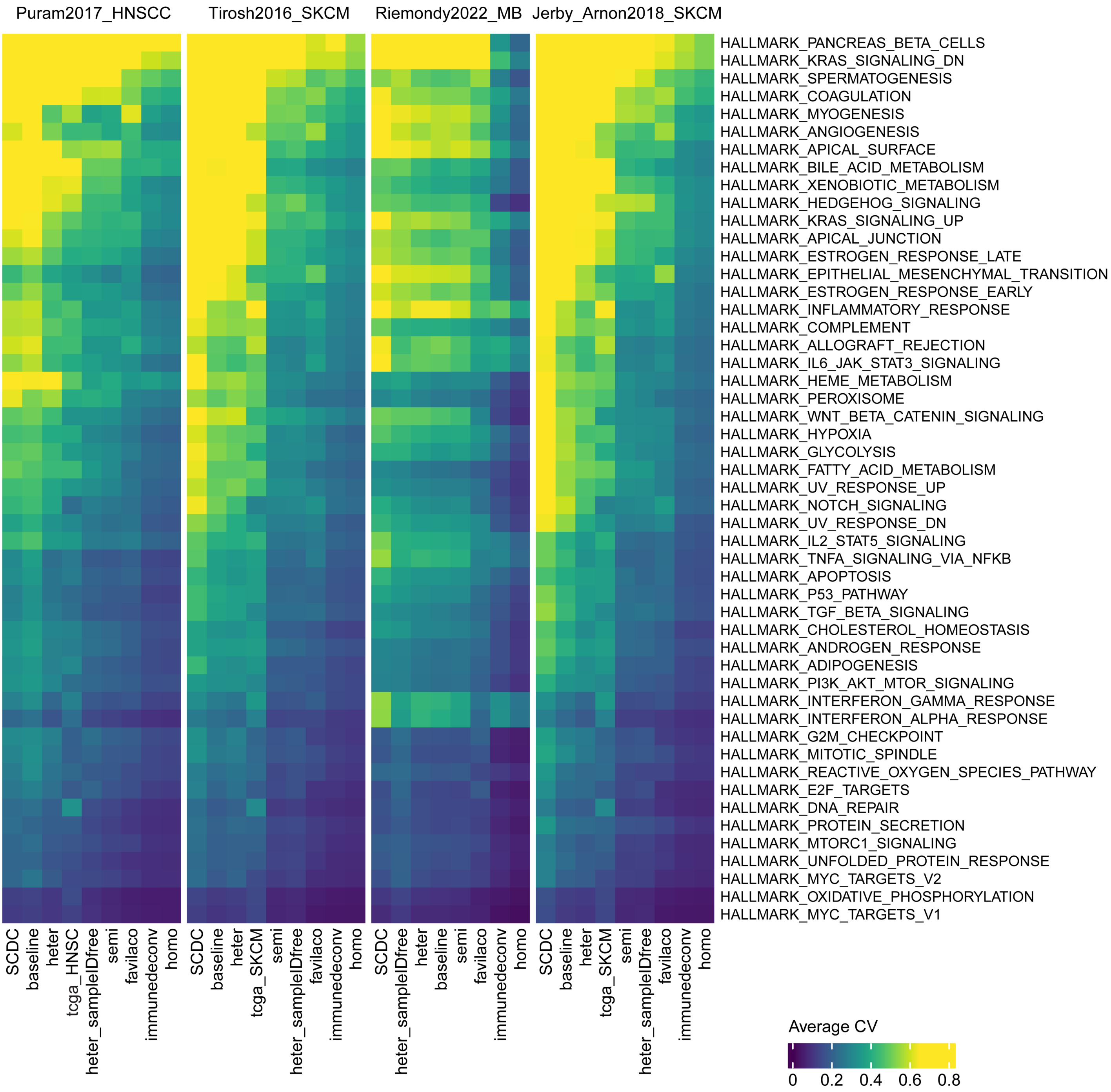

Fig S5. Comparison of different bulk simulation strategies: Variance in biological pathways

Heatmap comparing average coefficients of variance (CV) for genes from all 50 hallmark pathways, as an illustration of biological variations. The columns denote baseline and simulated bulk expression, along with real bulk expression profiles from TCGA when paired tumor type is available.

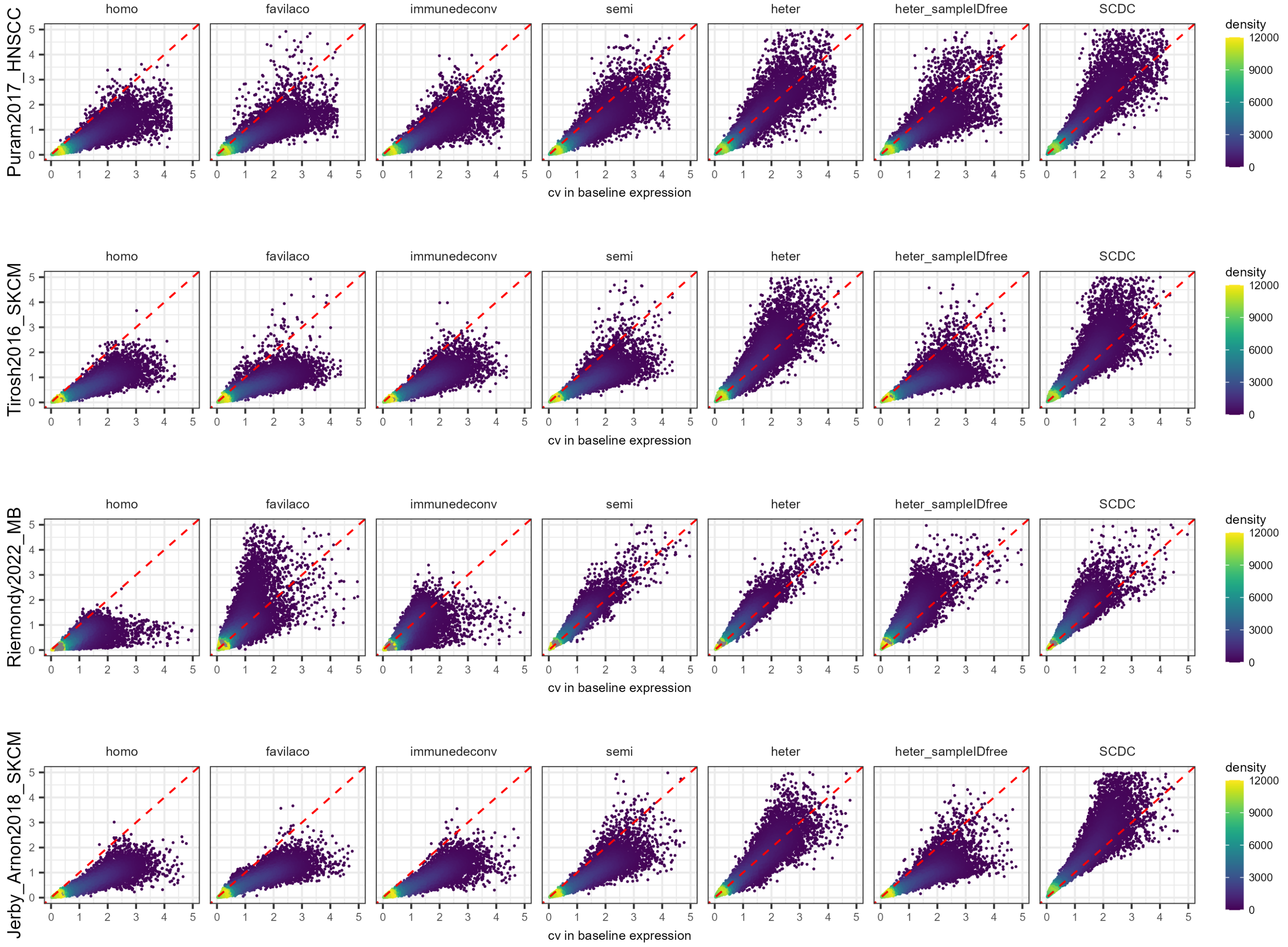

Fig S6. Comparison of different bulk simulation strategies: Scatter plot of variance comparison

Scatter plots comparing coefficients of variation (CV) for all genes between simulated and baseline expression, where the expression profiles are obtained using the same procedure as in FigS4.

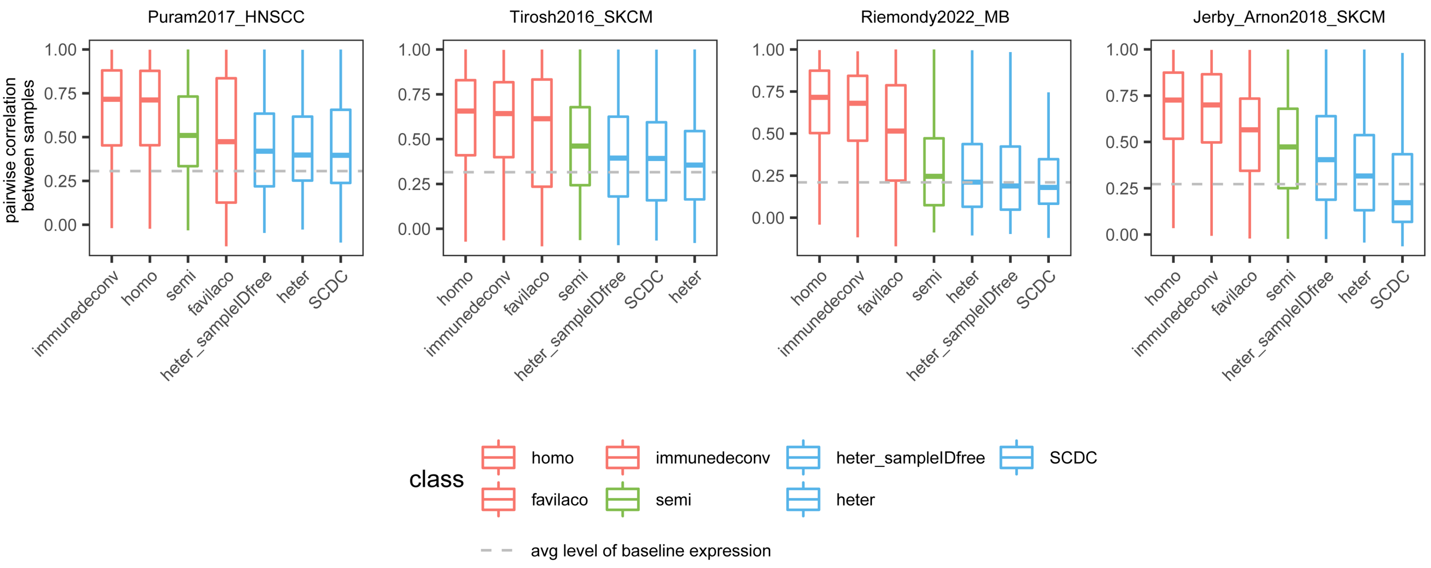

Fig S7. Comparison of different bulk simulation strategies: Distribution of sample-wise correlations
Boxplot comparing pairwise correlations between simulated bulk samples, with the dashed line indicating the average pairwise correlation in baseline expression.

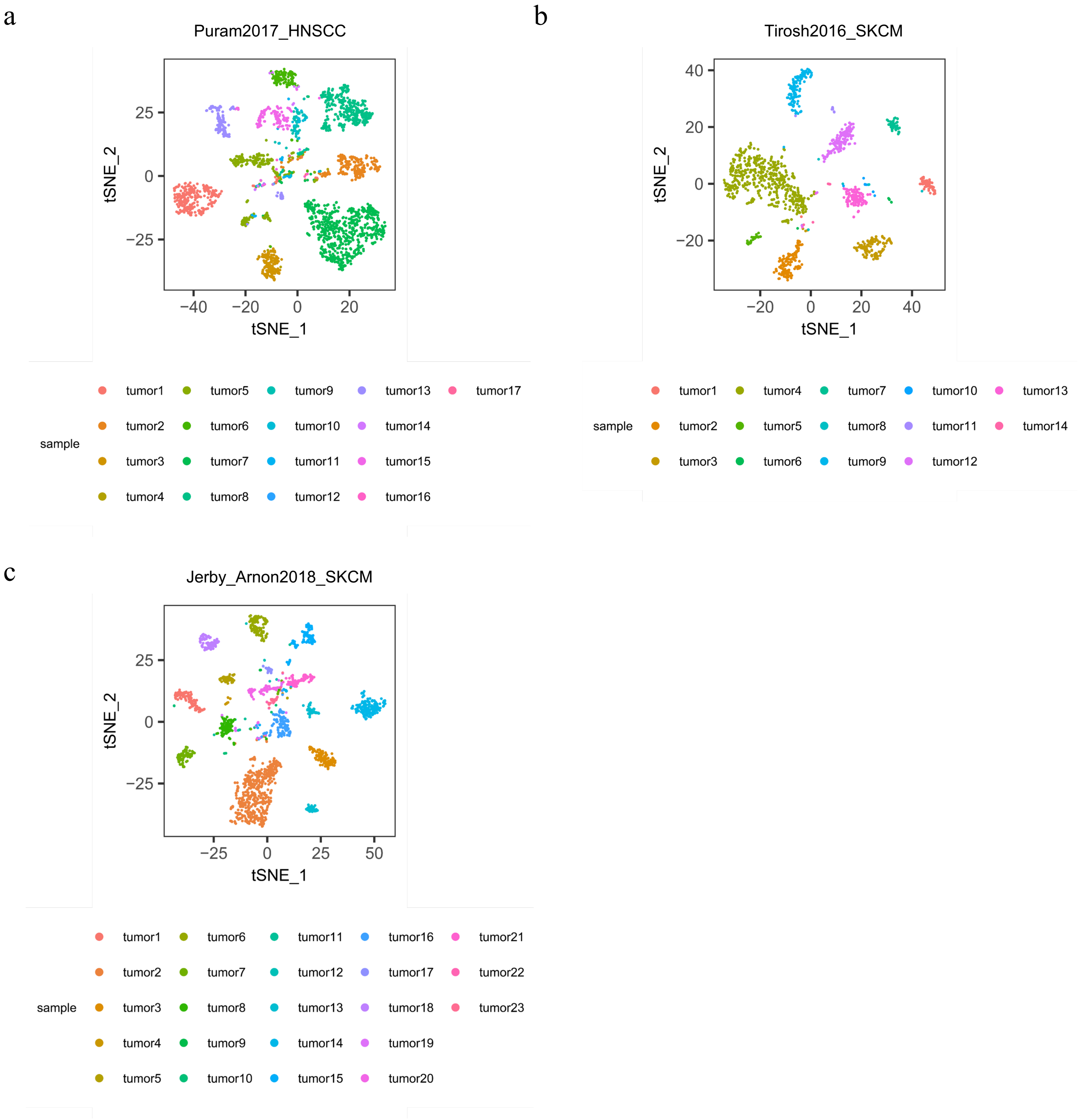

Fig S8. Heterogeneity in malignant cells
(a-c) tSNE plot of malignant cells from Puram2017_HNSCC dataset (n=2,539), Tirosh2016_SKCM dataset (n=1,310) and Jerby_Arnon2018_SKCM dataset (n=2,018), colored by patient identifiers.

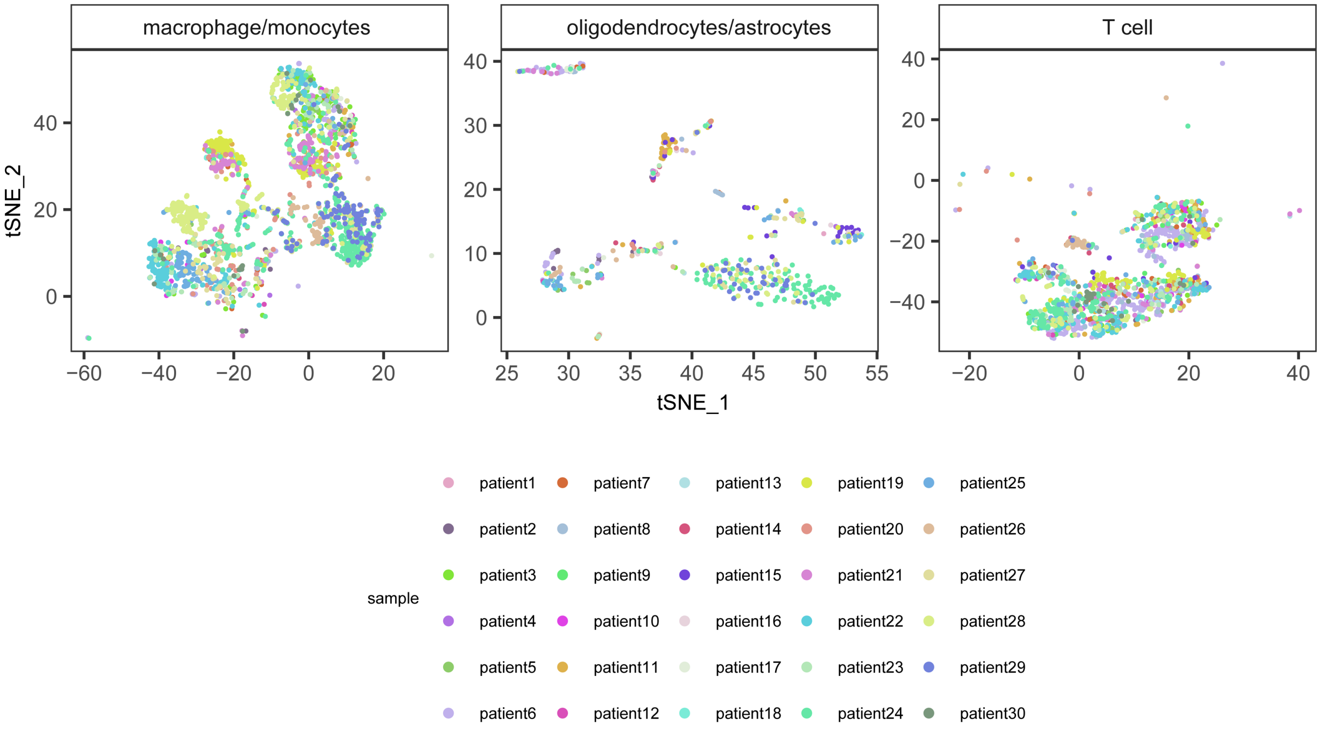

Fig S9. Heterogeneity in non-malignant cells

tSNE plot of non-malignant cells from the Riemondy2022_MB dataset, colored by patient identifiers. Representative cell-types with n > 500 cells are shown in this plot (macrophage/monocytes: n = 2107, oligodendrocytes/astrocytes: n= 539, T cell: n = 1344).

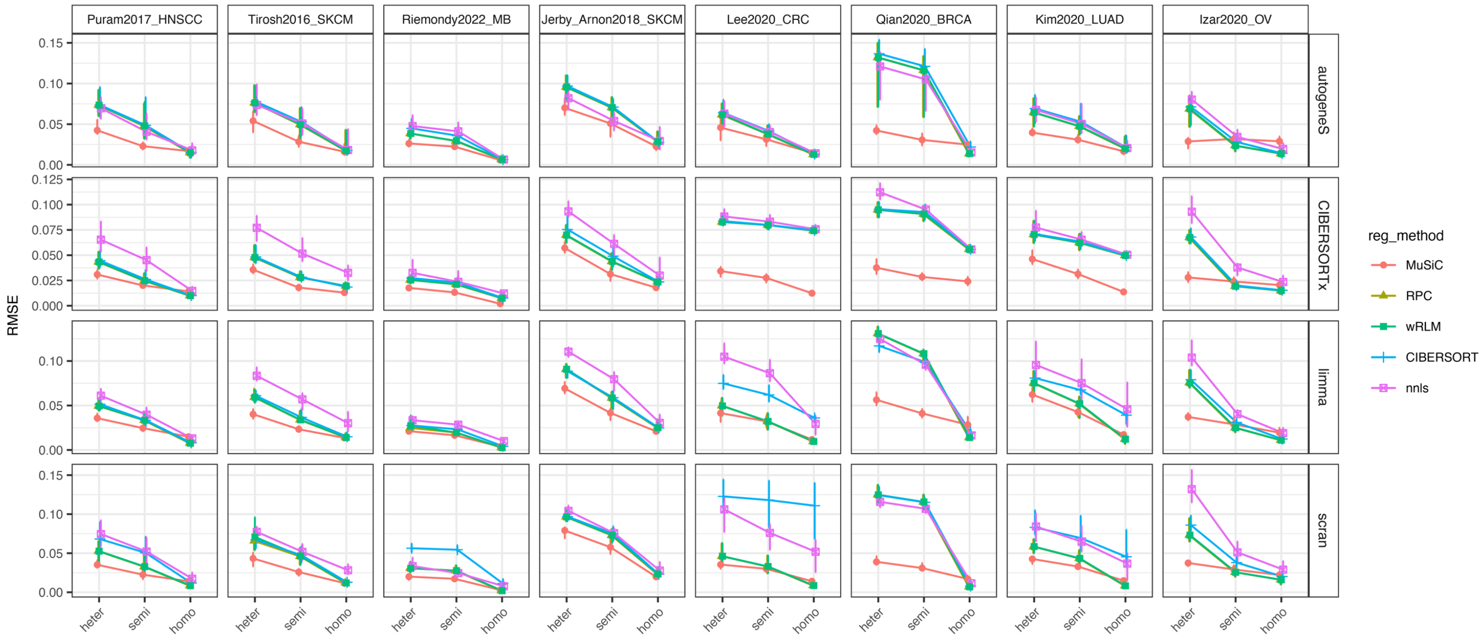

Fig S10. Overall RMSE for regression-based methods under various simulation settings

Line plot comparing performance of regression-based methods under various simulation strategies across eight different datasets. Performance is assessed using RMSE values, with lower RMSE corresponds to higher performance. The error bars indicate the min and max level of RMSE over 10 experimental repeats. Each row corresponds to a different method to generate the reference matrices.

a

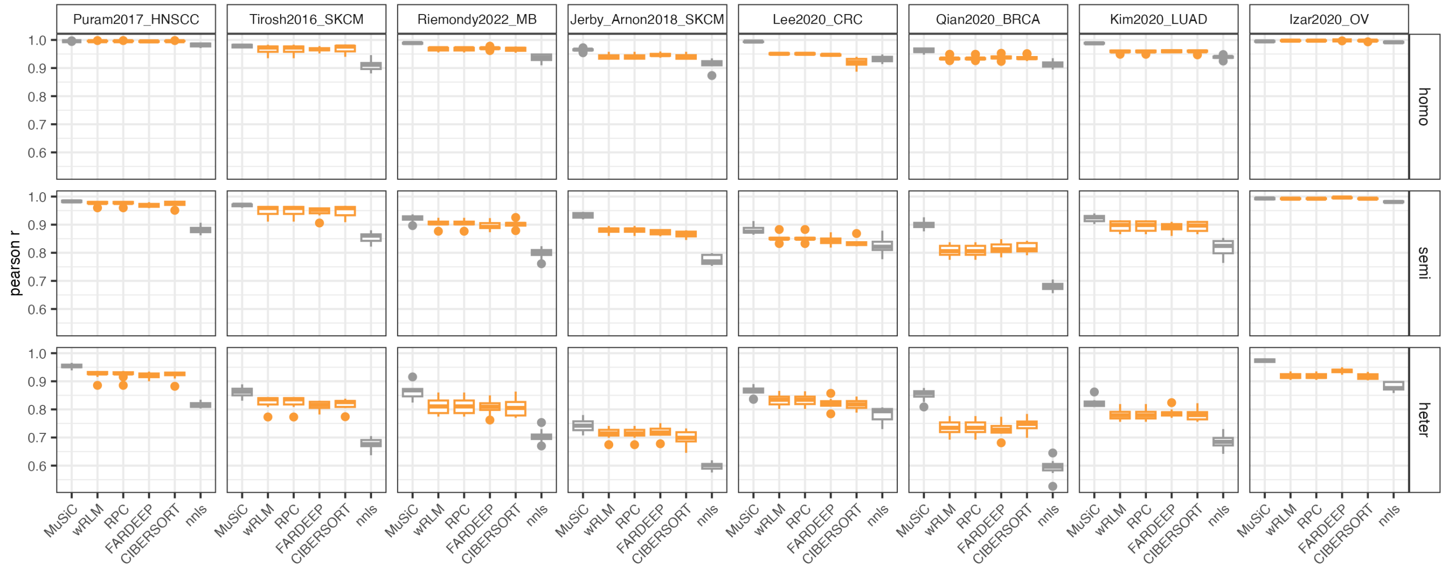

b

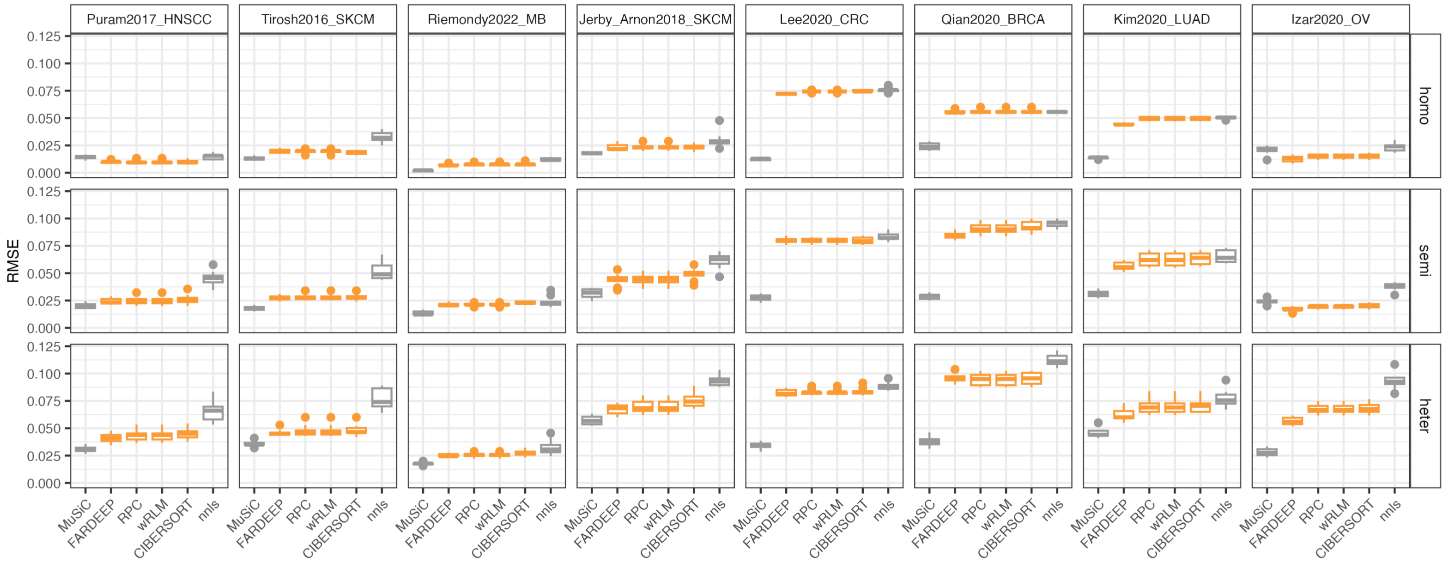

Fig S11. Robust regression methods show similar sensitivity to changes in heterogeneity levels

Boxplot showing the distribution of (a) Pearson correlation and (b) RMSE values of different regression methods across experimental repeats, using CIBSERSORTx derived reference matrix as input. Robust regression methods are highlighted in yellow.

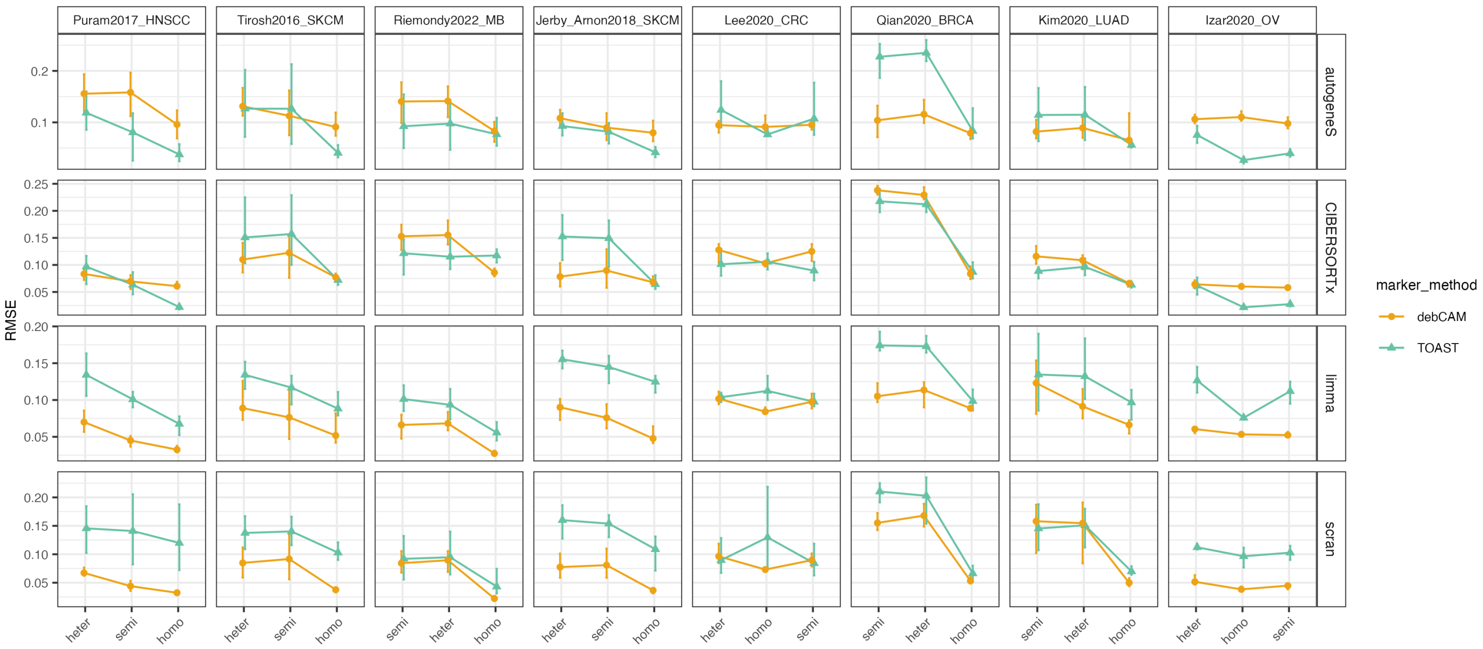

Fig S12. Overall RMSE for marker-based methods under various simulation settings
Line plot comparing performance of marker-based methods under various simulation strategies across eight different datasets. Performance is assessed using RMSE values, with lower RMSE corresponds to higher performance . The error bars indicate the min and max level of RMSE over 10 experimental repeats. Each row corresponds to a different method to generate the marker-list. Only marker-based methods with the sum-to-one constraint are included in this figure.

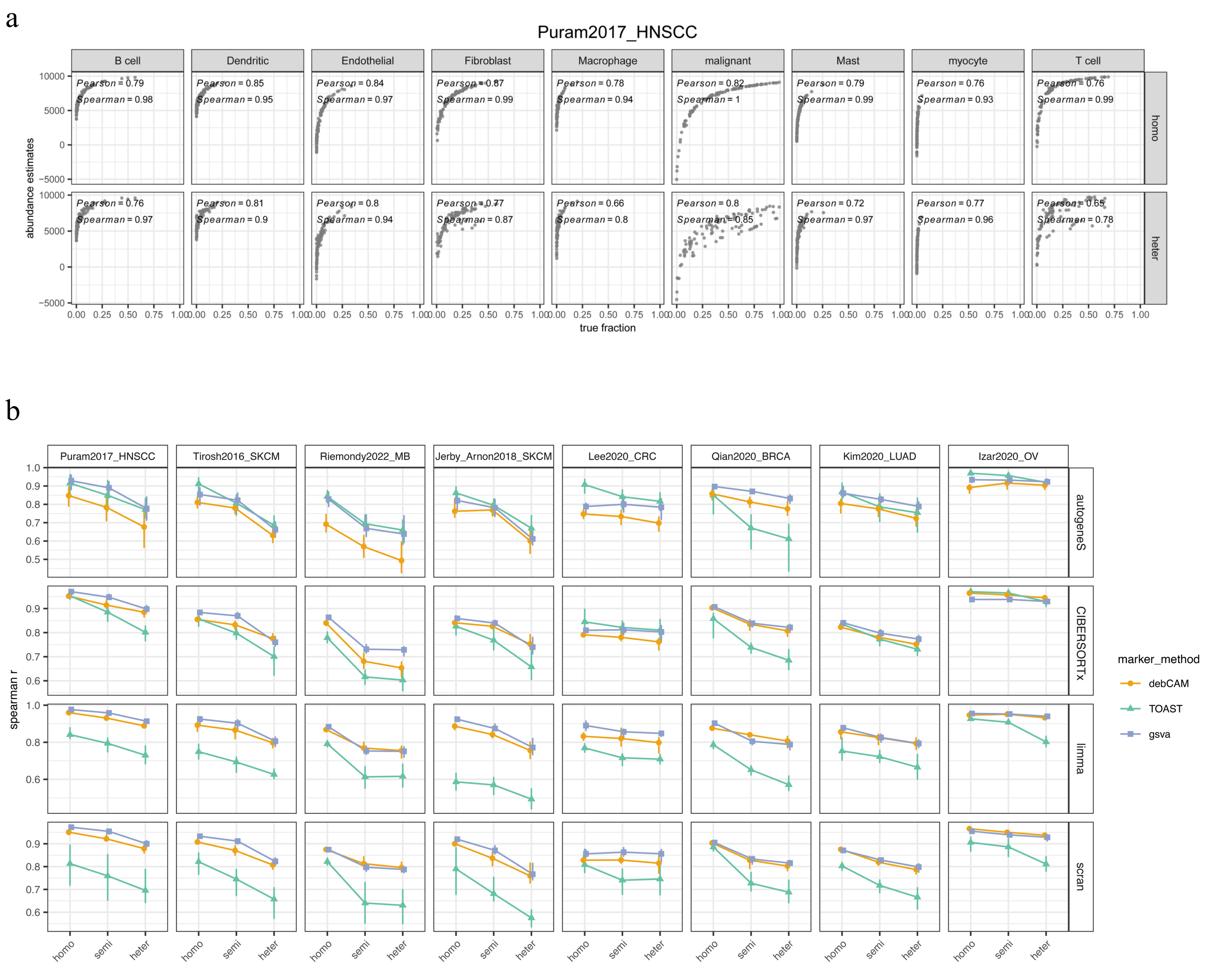

Fig S13. Gsva score estimates correlate non-linearly with ground truth fraction

(a) Scatter plot showing the performance of gsva under homo and heter simulation in one example dataset, with ground truth cell type fraction on x axis and the predicted scores on y axis. (b) Line plot showing the average spearman correlation of marker-based methods under different simulation strategies across eight different datasets, with the error bars correspond to the min and max level of spearman correlation over 10 experimental repeats.

a

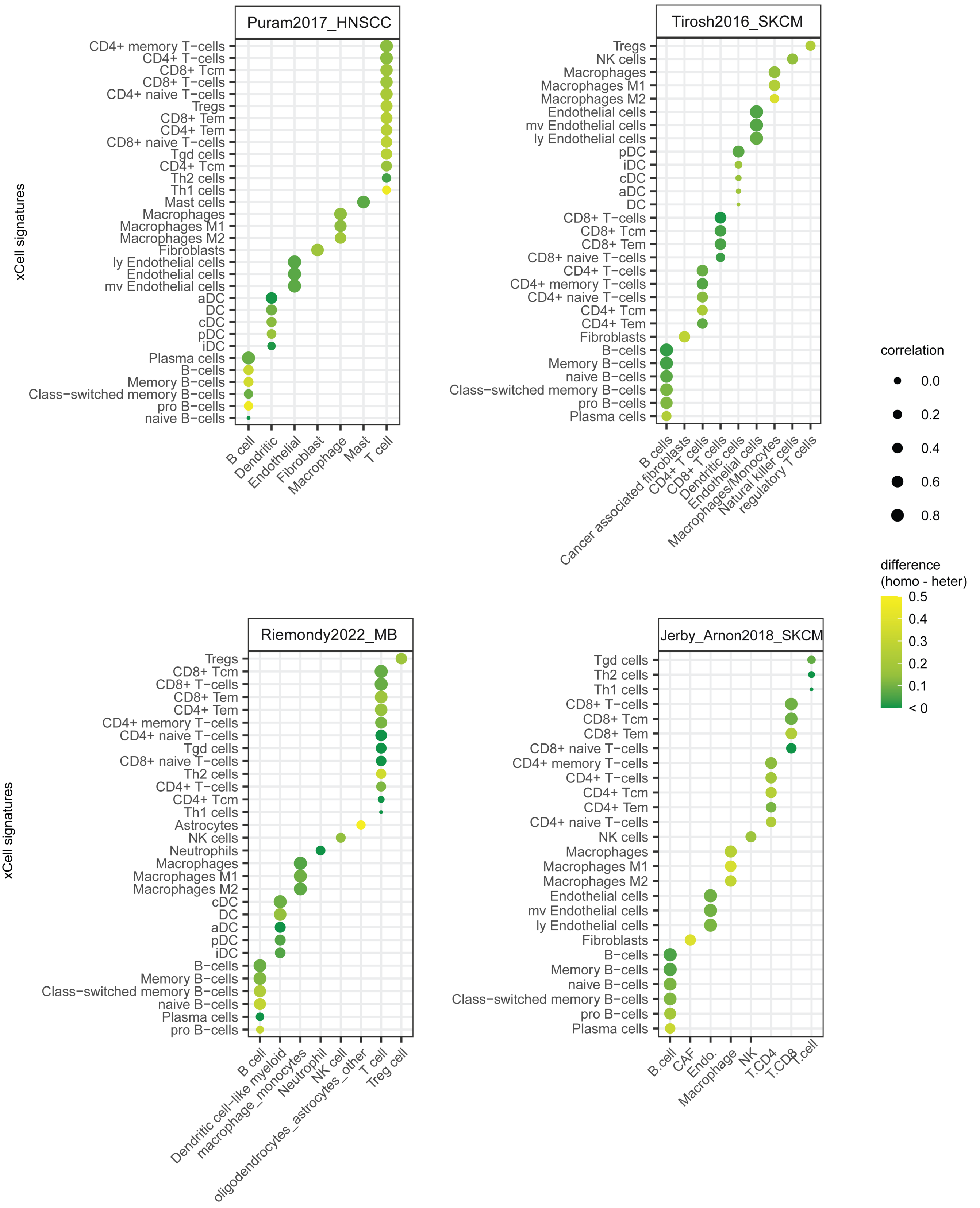

Puram2017_HNSCC
CD4+ memory T−cells
CD4+ T−cells
CD8+ Tcm
CD8+ T−cells
CD4+ naive T−cells
Tregs
CD8+ Tem
CD4+ Tem
CD8+ naive T−cells
Tgd cells
CD4+ Tcm
Th2 cells
Th1 cells
Mast cells
Macrophages
Macrophages M1
Macrophages M2
Fibroblasts
ly Endothelial cells
Endothelial cells
mv Endothelial cells
aDC
DC
cDC
pDC
iDC
Plasma cells
B−cells
Memory B−cells
Class−switched memory B−cells
pro B−cells
naive B−cells
B cell
Dendritic
Endothelial
Fibroblast
Macrophage
Mast
T cell
xCell signatures
Tirosh2016_SKCM
Tregs
NK cells
Macrophages
Macrophages M1
Macrophages M2
Endothelial cells
mv Endothelial cells
ly Endothelial cells
pDC
iDC
cDC
aDC
DC
CD8+ T−cells
CD8+ Tcm
CD8+ Tem
CD8+ naive T−cells
CD4+ T−cells
CD4+ memory T−cells
CD4+ naive T−cells
CD4+ Tcm
CD4+ Tem
Fibroblasts
B−cells
Memory B−cells
naive B−cells
Class−switched memory B−cells
pro B−cells
Plasma cells
B cells
Cancer associated fibroblasts
CD4+ T cells
CD8+ T cells
Dendritic cells
Endothelial cells
Macrophages/Monocytes
Natural killer cells
regulatory T cells
correlation
0.0
0.2
0.4
0.6
0.8
difference
(homo - heter)
0.5
0.4
0.3
0.2
0.1
< 0
Riemondy2022_MB
Tregs
CD8+ Tcm
CD8+ T−cells
CD8+ Tem
CD4+ Tem
CD4+ memory T−cells
CD4+ naive T−cells
Tgd cells
CD8+ naive T−cells
Th2 cells
CD4+ T−cells
CD4+ Tcm
Th1 cells
Astrocytes
NK cells
Neutrophils
Macrophages
Macrophages M1
Macrophages M2
cDC
DC
aDC
pDC
iDC
B−cells
Memory B−cells
Class−switched memory B−cells
naive B−cells
Plasma cells
pro B−cells
B cell
Dendritic cell-like myeloid
macrophage_monocytes
Neutrophil
NK cell
oligodendrocytes_astrocytes_other
T cell
Treg cell
xCell signatures
Jerby_Arnon2018_SKCM
Tgd cells
Th2 cells
Th1 cells
CD8+ T−cells
CD8+ Tcm
CD8+ Tem
CD8+ naive T−cells
CD4+ memory T−cells
CD4+ T−cells
CD4+ Tcm
CD4+ Tem
CD4+ naive T−cells
NK cells
Macrophages
Macrophages M1
Macrophages M2
Endothelial cells
mv Endothelial cells
ly Endothelial cells
Fibroblasts
B−cells
Memory B−cells
naive B−cells
Class−switched memory B−cells
pro B−cells
Plasma cells
B.cell
CAF
Endo.
Macrophage
NK
T.CD4
T.CD8
T.cell

b

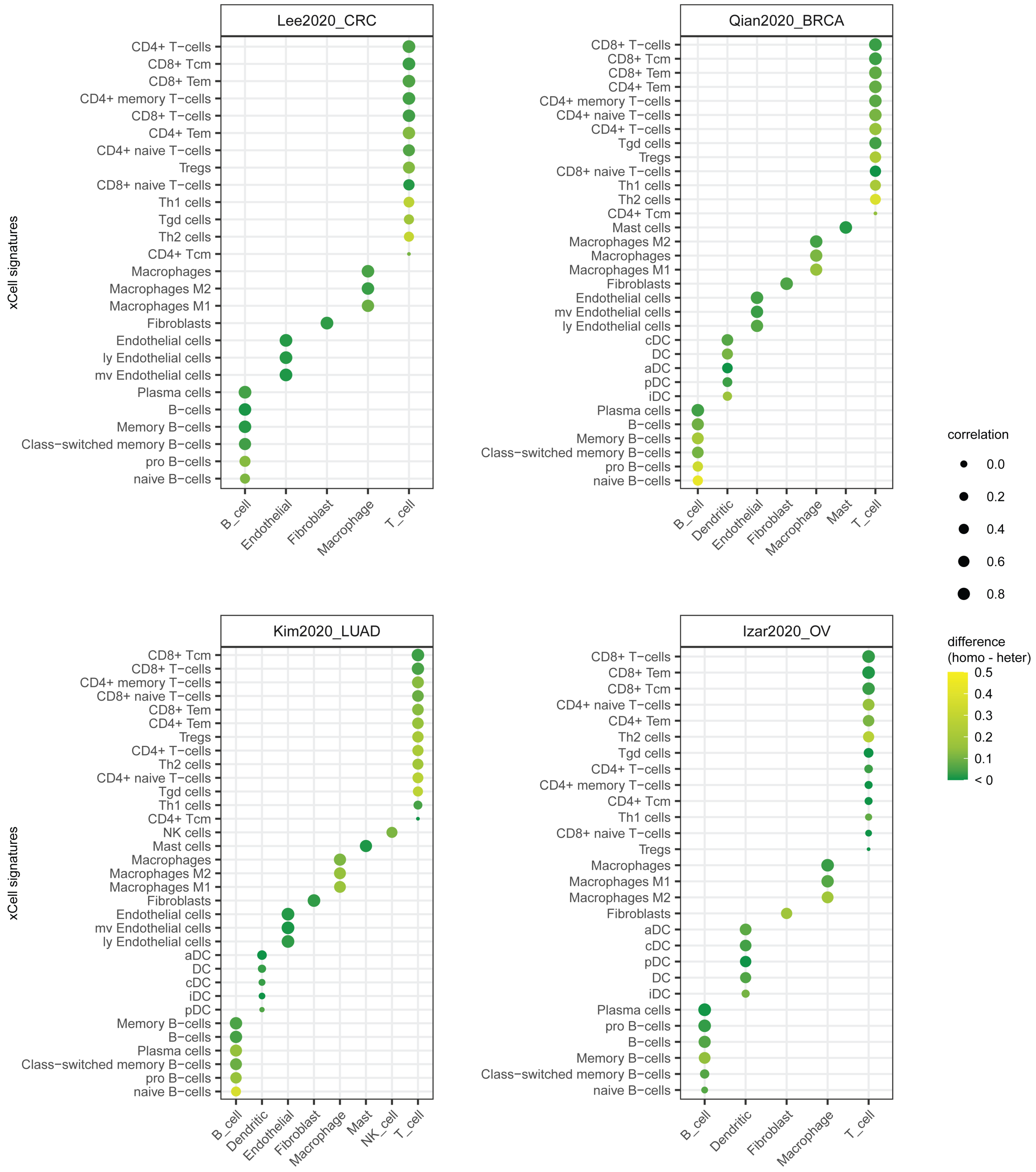

Fig S14. Performance of marker-based method with built-in signatures: Pearson correlation of xCell signatures Dot plot showing the performance of xCell scores in estimating cell type abundance, with ground truth cell-type fractions on the x axis, and the estimated xCell signatures on the y axis. The dot size indicates per-cell type Pearson correlation for heter simulated bulk samples, and dot color reflects the difference in Pearson correlation between homo and heter simulated samples. The xCell signatures are specifically mapped to relevant cell types.

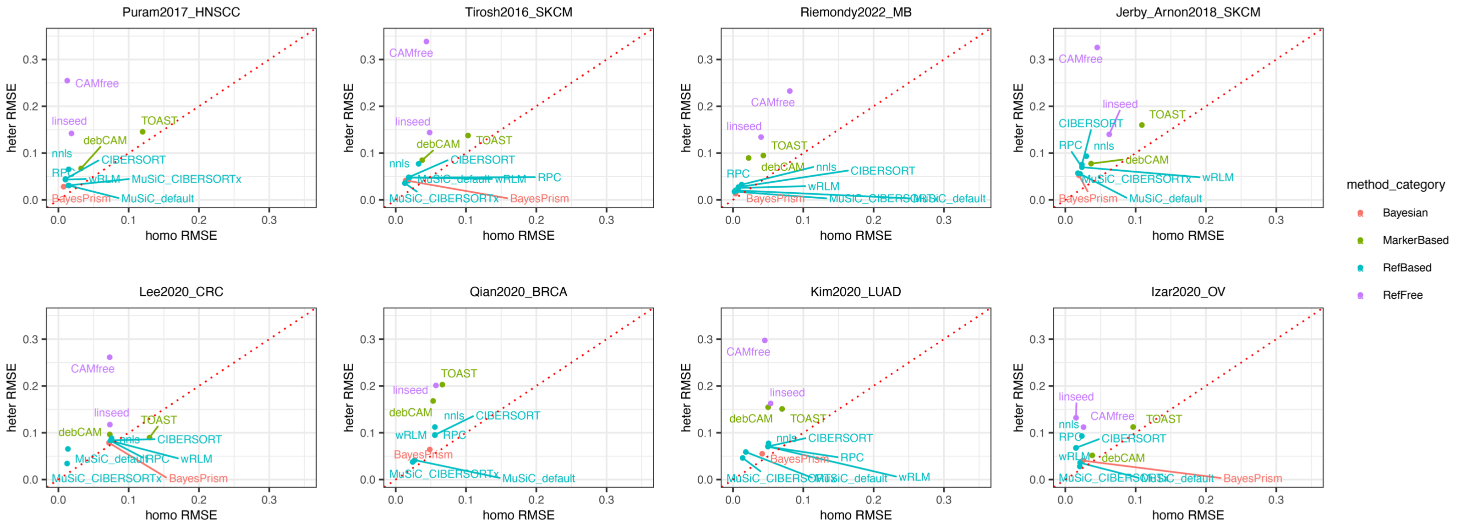

Fig S15. Overall RMSE comparison under homogeneous and heterogeneous conditions

Scatter plot comparing the average RMSE values of different deconvolution methods under homogeneous and heterogeneous simulations. Averages are calculated over 10 experimental repeats, with various colors representing different categories of deconvolution methods. All the regression-based methods are using CIBERSORTx derived reference matrix and all the marker-based methods are using scran derived markers, while "MuSiC_default" means the default MuSiC setting where all the genes are being used as input.

a

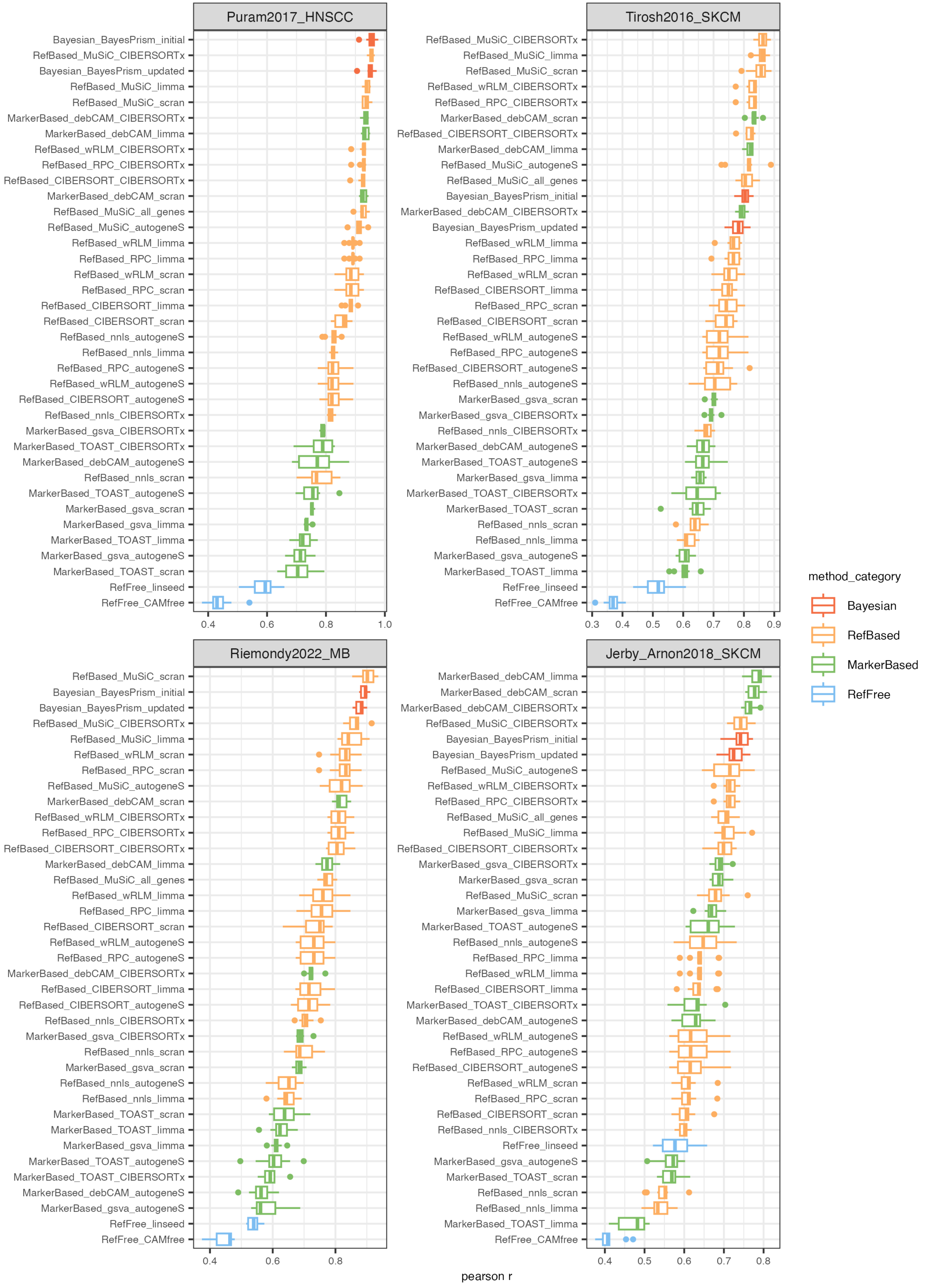

Puram2017_HNSCC
Bayesian_BayesPrism_initial
RefBased_MuSiC_CIBERSORTx
Bayesian_BayesPrism_updated
RefBased_MuSiC_limma
RefBased_MuSiC_scran
MarkerBased_debCAM_CIBERSORTx
MarkerBased_debCAM_limma
RefBased_wRLM_CIBERSORTx
RefBased_RPC_CIBERSORTx
RefBased_CIBERSORT_CIBERSORTx
MarkerBased_debCAM_scran
RefBased_MuSiC_all_genes
RefBased_MuSiC_autogeneS
RefBased_wRLM_limma
RefBased_RPC_limma
RefBased_wRLM_scran
RefBased_RPC_scran
RefBased_CIBERSORT_limma
RefBased_CIBERSORT_scran
RefBased_nnls_autogeneS
RefBased_nnls_limma
RefBased_RPC_autogeneS
RefBased_wRLM_autogeneS
RefBased_CIBERSORT_autogeneS
RefBased_nnls_CIBERSORTx
MarkerBased_gsva_CIBERSORTx
MarkerBased_TOAST_CIBERSORTx
MarkerBased_debCAM_autogeneS
RefBased_nnls_scran
MarkerBased_TOAST_autogeneS
MarkerBased_gsva_scran
MarkerBased_gsva_limma
MarkerBased_TOAST_limma
MarkerBased_gsva_autogeneS
MarkerBased_TOAST_scran
RefFree_linseed
RefFree_CAMfree
0.4
0.6
0.8
1.0
Tirosh2016_SKCM
RefBased_MuSiC_CIBERSORTx
RefBased_MuSiC_limma
RefBased_MuSiC_scran
RefBased_wRLM_CIBERSORTx
RefBased_RPC_CIBERSORTx
MarkerBased_debCAM_scran
RefBased_CIBERSORT_CIBERSORTx
MarkerBased_debCAM_limma
RefBased_MuSiC_autogeneS
RefBased_MuSiC_all_genes
Bayesian_BayesPrism_initial
MarkerBased_debCAM_CIBERSORTx
Bayesian_BayesPrism_updated
RefBased_wRLM_limma
RefBased_RPC_limma
RefBased_wRLM_scran
RefBased_CIBERSORT_limma
RefBased_RPC_scran
RefBased_CIBERSORT_scran
RefBased_wRLM_autogeneS
RefBased_RPC_autogeneS
RefBased_CIBERSORT_autogeneS
RefBased_nnls_autogeneS
MarkerBased_gsva_scran
MarkerBased_gsva_CIBERSORTx
RefBased_nnls_CIBERSORTx
MarkerBased_debCAM_autogeneS
MarkerBased_TOAST_autogeneS
MarkerBased_gsva_limma
MarkerBased_TOAST_CIBERSORTx
MarkerBased_TOAST_scran
RefBased_nnls_scran
RefBased_nnls_limma
MarkerBased_gsva_autogeneS
MarkerBased_TOAST_limma
RefFree_linseed
RefFree_CAMfree
0.3
0.4
0.5
0.6
0.7
0.8
0.9
Riemondy2022_MB
RefBased_MuSiC_scran
Bayesian_BayesPrism_initial
Bayesian_BayesPrism_updated
RefBased_MuSiC_CIBERSORTx
RefBased_MuSiC_limma
RefBased_wRLM_scran
RefBased_RPC_scran
RefBased_MuSiC_autogeneS
MarkerBased_debCAM_scran
RefBased_wRLM_CIBERSORTx
RefBased_RPC_CIBERSORTx
RefBased_CIBERSORT_CIBERSORTx
MarkerBased_debCAM_limma
RefBased_MuSiC_all_genes
RefBased_wRLM_limma
RefBased_RPC_limma
RefBased_CIBERSORT_scran
RefBased_wRLM_autogeneS
RefBased_RPC_autogeneS
MarkerBased_debCAM_CIBERSORTx
RefBased_CIBERSORT_limma
RefBased_CIBERSORT_autogeneS
RefBased_nnls_CIBERSORTx
MarkerBased_gsva_CIBERSORTx
RefBased_nnls_scran
MarkerBased_gsva_scran
RefBased_nnls_autogeneS
RefBased_nnls_limma
MarkerBased_TOAST_scran
MarkerBased_TOAST_limma
MarkerBased_gsva_limma
MarkerBased_TOAST_autogeneS
MarkerBased_TOAST_CIBERSORTx
MarkerBased_debCAM_autogeneS
MarkerBased_gsva_autogeneS
RefFree_linseed
RefFree_CAMfree
0.4
0.6
0.8
Jerby_Arnon2018_SKCM
MarkerBased_debCAM_limma
MarkerBased_debCAM_scran
MarkerBased_debCAM_CIBERSORTx
RefBased_MuSiC_CIBERSORTx
Bayesian_BayesPrism_initial
Bayesian_BayesPrism_updated
RefBased_MuSiC_autogeneS
RefBased_wRLM_CIBERSORTx
RefBased_RPC_CIBERSORTx
RefBased_MuSiC_all_genes
RefBased_MuSiC_limma
RefBased_CIBERSORT_CIBERSORTx
MarkerBased_gsva_CIBERSORTx
MarkerBased_gsva_scran
RefBased_MuSiC_scran
MarkerBased_gsva_limma
MarkerBased_TOAST_autogeneS
RefBased_nnls_autogeneS
RefBased_RPC_limma
RefBased_wRLM_limma
RefBased_CIBERSORT_limma
MarkerBased_TOAST_CIBERSORTx
MarkerBased_debCAM_autogeneS
RefBased_wRLM_autogeneS
RefBased_RPC_autogeneS
RefBased_CIBERSORT_autogeneS
RefBased_wRLM_scran
RefBased_RPC_scran
RefBased_CIBERSORT_scran
RefBased_nnls_CIBERSORTx
RefFree_linseed
MarkerBased_gsva_autogeneS
MarkerBased_TOAST_scran
RefBased_nnls_scran
RefBased_nnls_limma
MarkerBased_TOAST_limma
RefFree_CAMfree
0.4
0.5
0.6
0.7
0.8
pearson r
method_category
Bayesian
RefBased
MarkerBased
RefFree

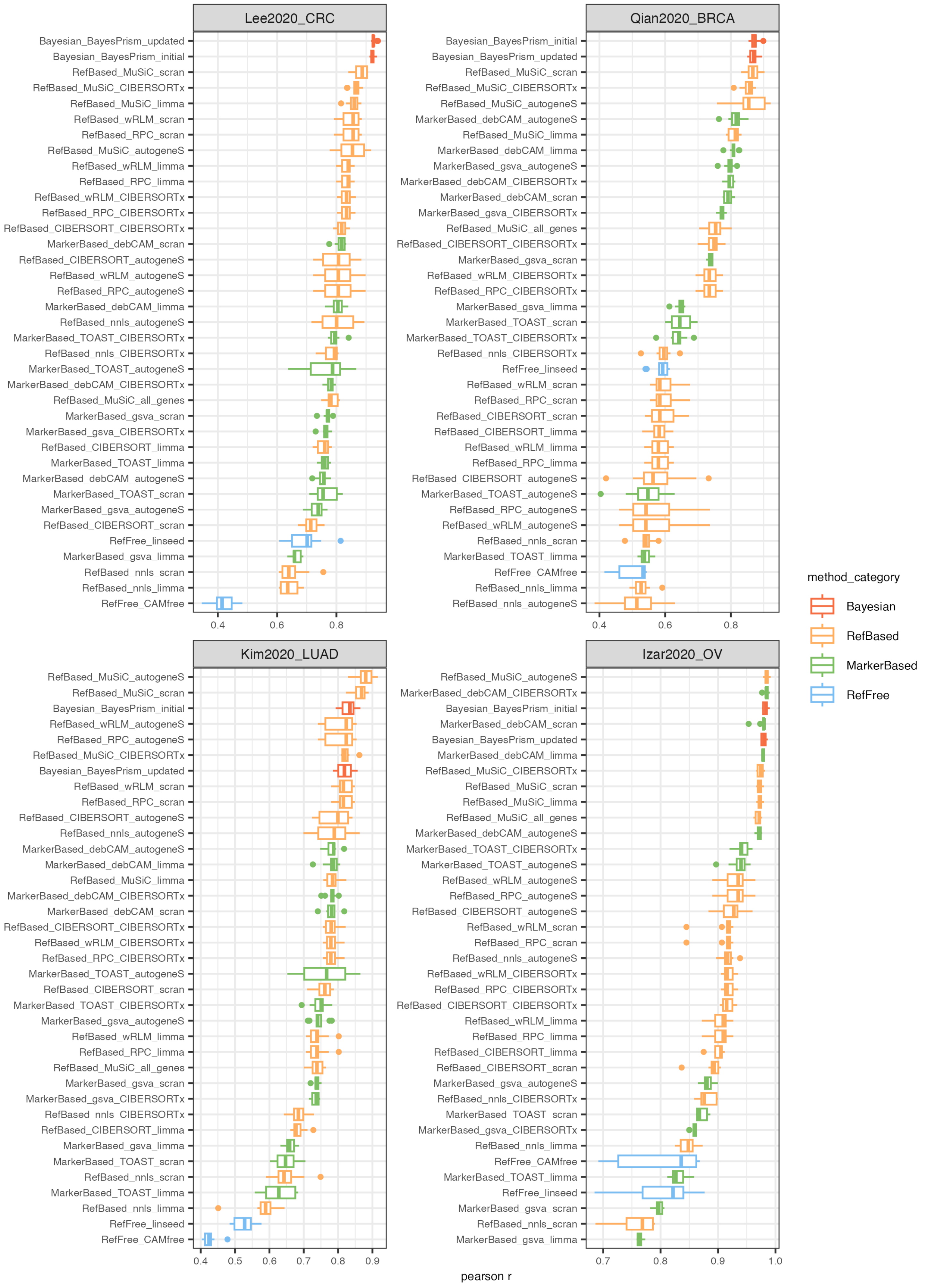

Lee2020_CRC
Qian2020_BRCA
Kim2020_LUAD
Izar2020_OV
pearson r
method_category
Bayesian
RefBased
MarkerBased
RefFree

b

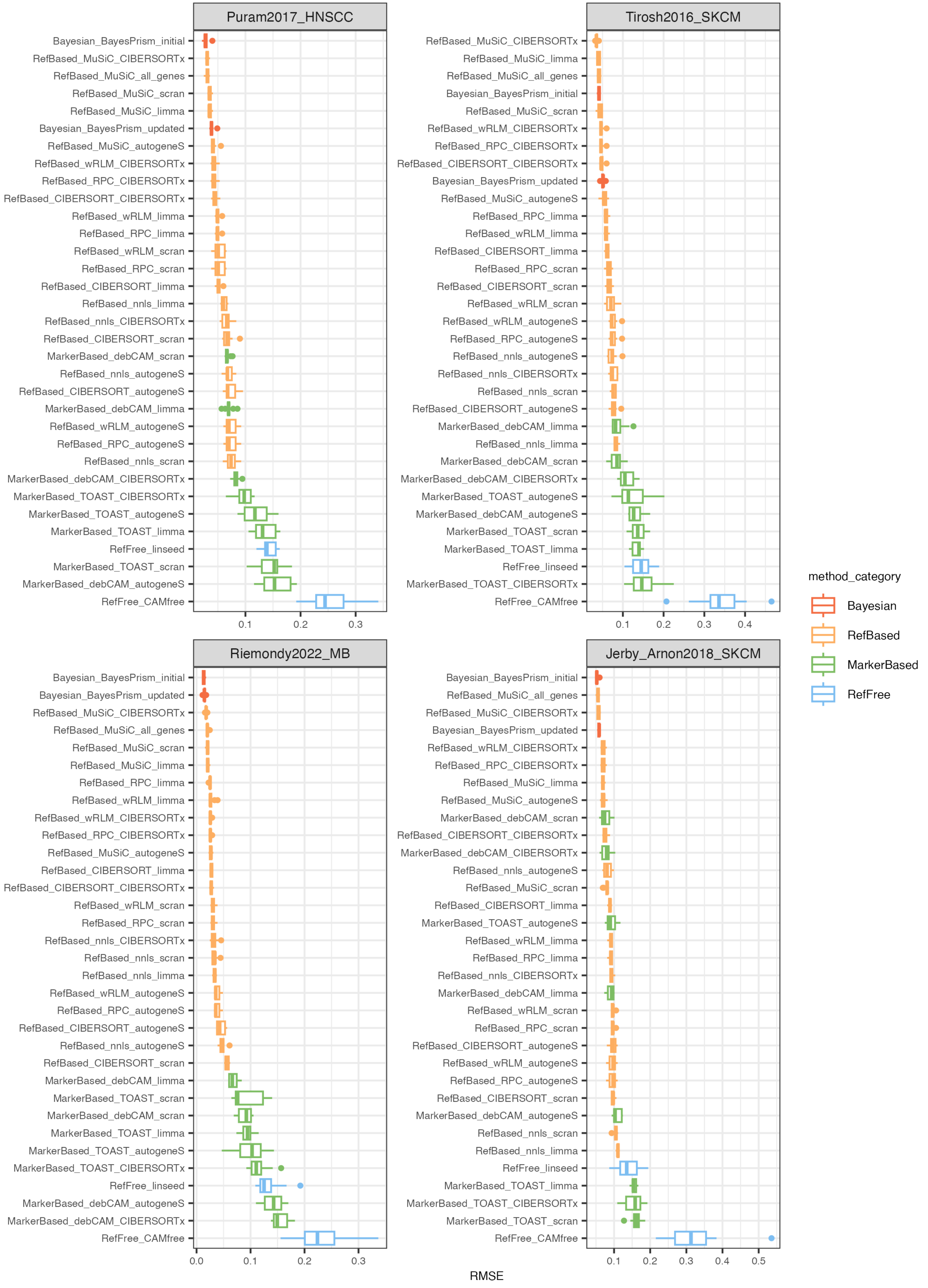

Puram2017_HNSCC
Bayesian_BayesPrism_initial
RefBased_MuSiC_CIBERSORTx
RefBased_MuSiC_all_genes
RefBased_MuSiC_scran
RefBased_MuSiC_limma
Bayesian_BayesPrism_updated
RefBased_MuSiC_autogeneS
RefBased_wRLM_CIBERSORTx
RefBased_RPC_CIBERSORTx
RefBased_CIBERSORT_CIBERSORTx
RefBased_wRLM_limma
RefBased_RPC_limma
RefBased_wRLM_scran
RefBased_RPC_scran
RefBased_CIBERSORT_limma
RefBased_nnls_limma
RefBased_nnls_CIBERSORTx
RefBased_CIBERSORT_scran
MarkerBased_debCAM_scran
RefBased_nnls_autogeneS
RefBased_CIBERSORT_autogeneS
MarkerBased_debCAM_limma
RefBased_wRLM_autogeneS
RefBased_RPC_autogeneS
RefBased_nnls_scran
MarkerBased_debCAM_CIBERSORTx
MarkerBased_TOAST_CIBERSORTx
MarkerBased_TOAST_autogeneS
MarkerBased_TOAST_limma
RefFree_linseed
MarkerBased_TOAST_scran
MarkerBased_debCAM_autogeneS
RefFree_CAMfree
0.1
0.2
0.3
Tirosh2016_SKCM
RefBased_MuSiC_CIBERSORTx
RefBased_MuSiC_limma
RefBased_MuSiC_all_genes
Bayesian_BayesPrism_initial
RefBased_MuSiC_scran
RefBased_wRLM_CIBERSORTx
RefBased_RPC_CIBERSORTx
RefBased_CIBERSORT_CIBERSORTx
Bayesian_BayesPrism_updated
RefBased_MuSiC_autogeneS
RefBased_RPC_limma
RefBased_wRLM_limma
RefBased_CIBERSORT_limma
RefBased_RPC_scran
RefBased_CIBERSORT_scran
RefBased_wRLM_scran
RefBased_wRLM_autogeneS
RefBased_RPC_autogeneS
RefBased_nnls_autogeneS
RefBased_nnls_CIBERSORTx
RefBased_nnls_scran
RefBased_CIBERSORT_autogeneS
MarkerBased_debCAM_limma
RefBased_nnls_limma
MarkerBased_debCAM_scran
MarkerBased_debCAM_CIBERSORTx
MarkerBased_TOAST_autogeneS
MarkerBased_debCAM_autogeneS
MarkerBased_TOAST_scran
MarkerBased_TOAST_limma
RefFree_linseed
MarkerBased_TOAST_CIBERSORTx
RefFree_CAMfree
0.1
0.2
0.3
0.4
method_category
Bayesian
RefBased
MarkerBased
RefFree
Riemondy2022_MB
Bayesian_BayesPrism_initial
Bayesian_BayesPrism_updated
RefBased_MuSiC_CIBERSORTx
RefBased_MuSiC_all_genes
RefBased_MuSiC_scran
RefBased_MuSiC_limma
RefBased_RPC_limma
RefBased_wRLM_limma
RefBased_wRLM_CIBERSORTx
RefBased_RPC_CIBERSORTx
RefBased_MuSiC_autogeneS
RefBased_CIBERSORT_limma
RefBased_CIBERSORT_CIBERSORTx
RefBased_wRLM_scran
RefBased_RPC_scran
RefBased_nnls_CIBERSORTx
RefBased_nnls_scran
RefBased_nnls_limma
RefBased_wRLM_autogeneS
RefBased_RPC_autogeneS
RefBased_CIBERSORT_autogeneS
RefBased_nnls_autogeneS
RefBased_CIBERSORT_scran
MarkerBased_debCAM_limma
MarkerBased_TOAST_scran
MarkerBased_debCAM_scran
MarkerBased_TOAST_limma
MarkerBased_TOAST_autogeneS
MarkerBased_TOAST_CIBERSORTx
RefFree_linseed
MarkerBased_debCAM_autogeneS
MarkerBased_debCAM_CIBERSORTx
RefFree_CAMfree
0.0
0.1
0.2
0.3
Jerby_Arnon2018_SKCM
Bayesian_BayesPrism_initial
RefBased_MuSiC_all_genes
RefBased_MuSiC_CIBERSORTx
Bayesian_BayesPrism_updated
RefBased_wRLM_CIBERSORTx
RefBased_RPC_CIBERSORTx
RefBased_MuSiC_limma
RefBased_MuSiC_autogeneS
MarkerBased_debCAM_scran
RefBased_CIBERSORT_CIBERSORTx
MarkerBased_debCAM_CIBERSORTx
RefBased_nnls_autogeneS
RefBased_MuSiC_scran
RefBased_CIBERSORT_limma
MarkerBased_TOAST_autogeneS
RefBased_wRLM_limma
RefBased_RPC_limma
RefBased_nnls_CIBERSORTx
MarkerBased_debCAM_limma
RefBased_wRLM_scran
RefBased_RPC_scran
RefBased_CIBERSORT_autogeneS
RefBased_wRLM_autogeneS
RefBased_RPC_autogeneS
RefBased_CIBERSORT_scran
MarkerBased_debCAM_autogeneS
RefBased_nnls_scran
RefBased_nnls_limma
RefFree_linseed
MarkerBased_TOAST_limma
MarkerBased_TOAST_CIBERSORTx
MarkerBased_TOAST_scran
RefFree_CAMfree
0.1
0.2
0.3
0.4
0.5
RMSE

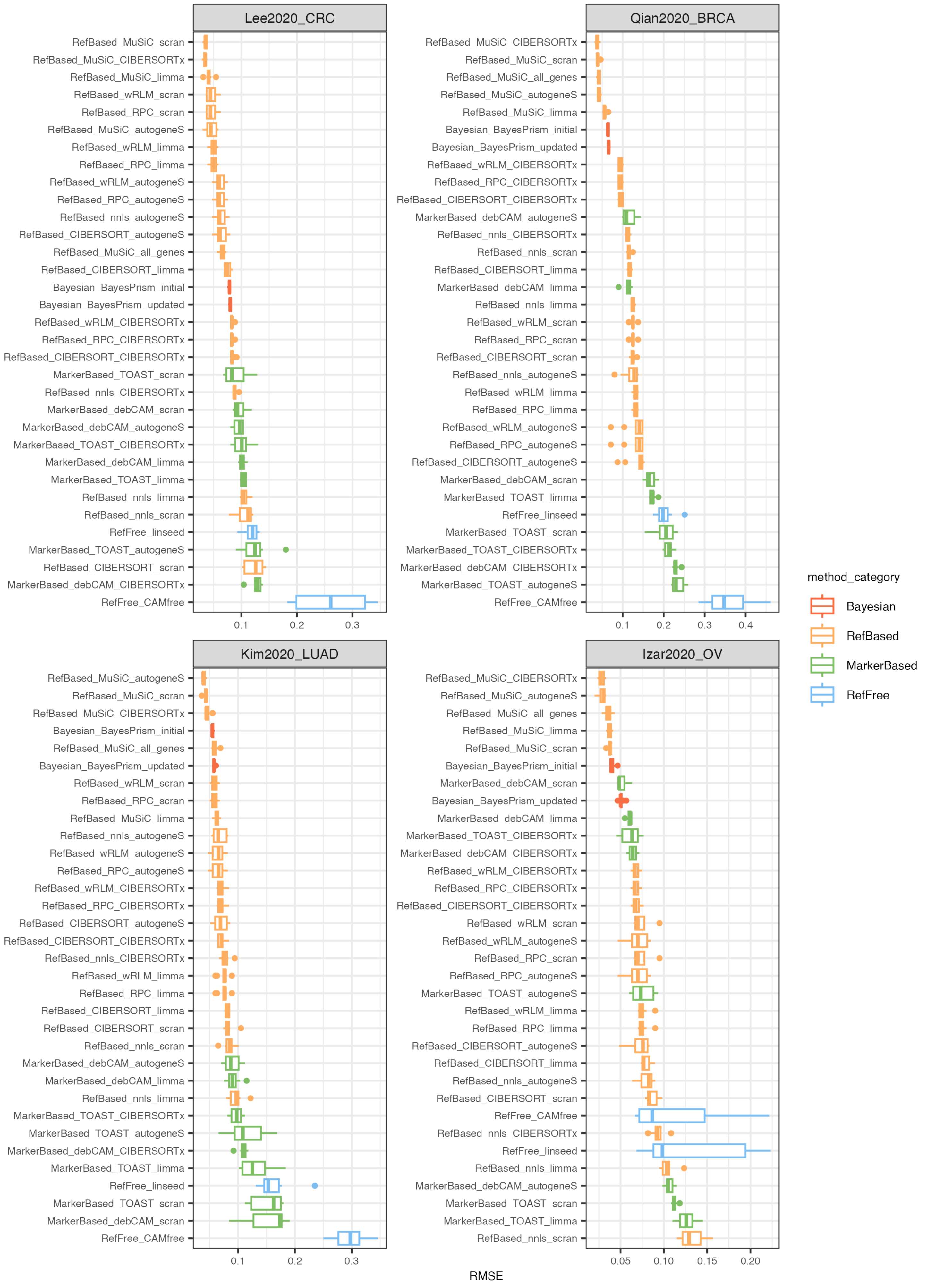
Lee2020_CRC
RefBased_MuSiC_scran
RefBased_MuSiC_CIBERSORTx
RefBased_MuSiC_limma
RefBased_wRLM_scran
RefBased_RPC_scran
RefBased_MuSiC_autogeneS
RefBased_wRLM_limma
RefBased_RPC_limma
RefBased_wRLM_autogeneS
RefBased_RPC_autogeneS
RefBased_nnls_autogeneS
RefBased_CIBERSORT_autogeneS
RefBased_MuSiC_all_genes
RefBased_CIBERSORT_limma
Bayesian_BayesPrism_initial
Bayesian_BayesPrism_updated
RefBased_wRLM_CIBERSORTx
RefBased_RPC_CIBERSORTx
RefBased_CIBERSORT_CIBERSORTx
MarkerBased_TOAST_scran
RefBased_nnls_CIBERSORTx
MarkerBased_debCAM_scran
MarkerBased_debCAM_autogeneS
MarkerBased_TOAST_CIBERSORTx
MarkerBased_debCAM_limma
MarkerBased_TOAST_limma
RefBased_nnls_limma
RefBased_nnls_scran
RefFree_linseed
MarkerBased_TOAST_autogeneS
RefBased_CIBERSORT_scran
MarkerBased_debCAM_CIBERSORTx
RefFree_CAMfree
0.1
0.2
0.3
Qian2020_BRCA
RefBased_MuSiC_CIBERSORTx
RefBased_MuSiC_scran
RefBased_MuSiC_all_genes
RefBased_MuSiC_autogeneS
RefBased_MuSiC_limma
Bayesian_BayesPrism_initial
Bayesian_BayesPrism_updated
RefBased_wRLM_CIBERSORTx
RefBased_RPC_CIBERSORTx
RefBased_CIBERSORT_CIBERSORTx
MarkerBased_debCAM_autogeneS
RefBased_nnls_CIBERSORTx
RefBased_nnls_scran
RefBased_CIBERSORT_limma
MarkerBased_debCAM_limma
RefBased_nnls_limma
RefBased_wRLM_scran
RefBased_RPC_scran
RefBased_CIBERSORT_scran
RefBased_nnls_autogeneS
RefBased_wRLM_limma
RefBased_RPC_limma
RefBased_wRLM_autogeneS
RefBased_RPC_autogeneS
RefBased_CIBERSORT_autogeneS
MarkerBased_debCAM_scran
MarkerBased_TOAST_limma
RefFree_linseed
MarkerBased_TOAST_scran
MarkerBased_TOAST_CIBERSORTx
MarkerBased_debCAM_CIBERSORTx
MarkerBased_TOAST_autogeneS
RefFree_CAMfree
0.1
0.2
0.3
0.4
Kim2020_LUAD
RefBased_MuSiC_autogeneS
RefBased_MuSiC_scran
RefBased_MuSiC_CIBERSORTx
Bayesian_BayesPrism_initial
RefBased_MuSiC_all_genes
Bayesian_BayesPrism_updated
RefBased_wRLM_scran
RefBased_RPC_scran
RefBased_MuSiC_limma
RefBased_nnls_autogeneS
RefBased_wRLM_autogeneS
RefBased_RPC_autogeneS
RefBased_wRLM_CIBERSORTx
RefBased_RPC_CIBERSORTx
RefBased_CIBERSORT_autogeneS
RefBased_CIBERSORT_CIBERSORTx
RefBased_nnls_CIBERSORTx
RefBased_wRLM_limma
RefBased_RPC_limma
RefBased_CIBERSORT_limma
RefBased_CIBERSORT_scran
RefBased_nnls_scran
MarkerBased_debCAM_autogeneS
MarkerBased_debCAM_limma
RefBased_nnls_limma
MarkerBased_TOAST_CIBERSORTx
MarkerBased_TOAST_autogeneS
MarkerBased_debCAM_CIBERSORTx
MarkerBased_TOAST_limma
RefFree_linseed
MarkerBased_TOAST_scran
MarkerBased_debCAM_scran
RefFree_CAMfree
0.1
0.2
0.3
Izar2020_OV
RefBased_MuSiC_CIBERSORTx
RefBased_MuSiC_autogeneS
RefBased_MuSiC_all_genes
RefBased_MuSiC_limma
RefBased_MuSiC_scran
Bayesian_BayesPrism_initial
MarkerBased_debCAM_scran
Bayesian_BayesPrism_updated
MarkerBased_debCAM_limma
MarkerBased_TOAST_CIBERSORTx
MarkerBased_debCAM_CIBERSORTx
RefBased_wRLM_CIBERSORTx
RefBased_RPC_CIBERSORTx
RefBased_CIBERSORT_CIBERSORTx
RefBased_wRLM_scran
RefBased_wRLM_autogeneS
RefBased_RPC_scran
RefBased_RPC_autogeneS
MarkerBased_TOAST_autogeneS
RefBased_wRLM_limma
RefBased_RPC_limma
RefBased_CIBERSORT_autogeneS
RefBased_CIBERSORT_limma
RefBased_nnls_autogeneS
RefBased_CIBERSORT_scran
RefFree_CAMfree
RefBased_nnls_CIBERSORTx
RefFree_linseed
RefBased_nnls_limma
MarkerBased_debCAM_autogeneS
MarkerBased_TOAST_scran
MarkerBased_TOAST_limma
RefBased_nnls_scran
0.05
0.10
0.15
0.20
RMSE
method_category
Bayesian
RefBased
MarkerBased
RefFree

Fig S16. Detailed performance comparison under heterogeneous simulation

Boxplot comparing the performance of all deconvolution methods under heterogeneous simulation strategy, as evaluated by (a) average Pearson correlation and (b) average RMSE values over 10 experimental repeats. The boxes are colored by different categories of deconvolution methods and the deconvolution methods are named using the format ‘‘deconvolution category_’ + ‘deconvolution method’ format, followed by reference generation methods or updating methods when applicable.

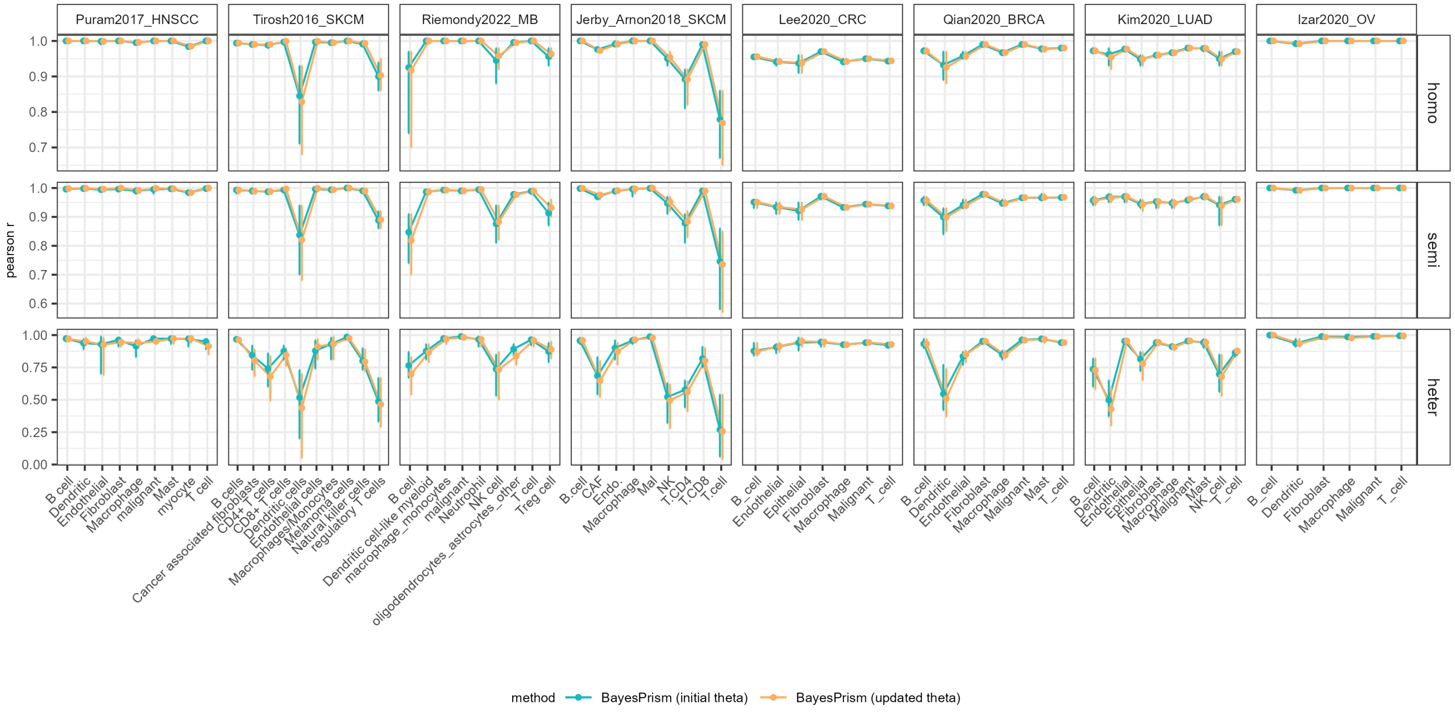

Fig S17. BayesPrism using initial vs updated reference : Per cell-type Pearson correlation comparison

Lineplot comparing cell-type level performance for BayesPrism with initial reference (using update.gibbs=FALSE) and updated reference (using update.gibbs=TRUE), with error bar indicating the min and max level of Pearson correlation over 10 experimental repeats.

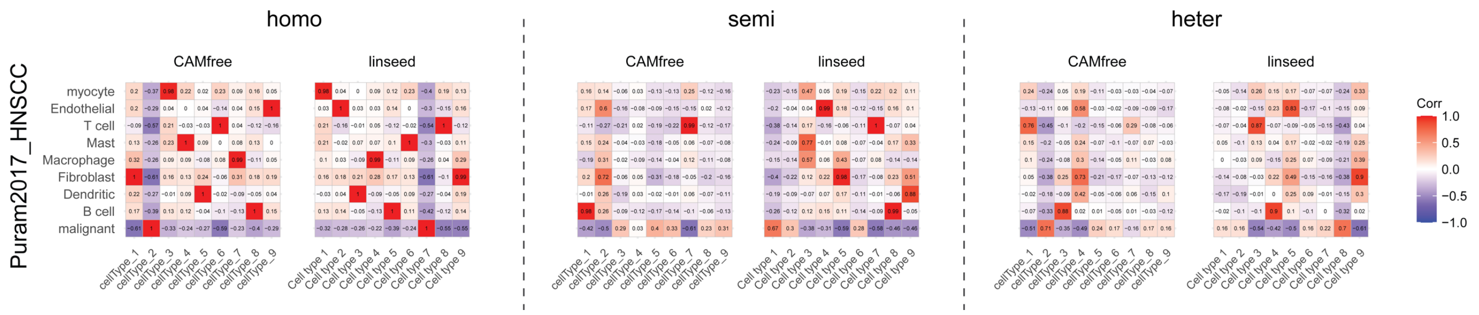

Fig S18. Pairwise correlation between fraction estimates and ground truth fractions facilitates cell-type mapping in reference-free methods

Heatmap depicting pairwise correlation between cell-type fractions and reference-free identified cell-type fractions under different simulation strategies from an example dataset.
