# Supplementary material for: Heterogeneous pseudobulk simulation enables realistic benchmarking of cell-type deconvolution methods
Source: Genome Biol. 2024 Jul 1;25:169. doi: 10.1186/s13059-024-03292-w (PMC11218230; doi:10.1186/s13059-024-03292-w)
Supplement: Supplementary file 3 — Additional file 3. Review history. [file 13059_2024_3292_MOESM3_ESM.docx]

**1^st^ round**

**Reviewer 1**

This manuscript presents a scheme for generating pseudobulk RNA samples for the purpose of benchmarking cell type deconvolution methods. They demonstrate that using the more common, simplistic approach (randomly selecting cells from each cell type to compose a pseudobulk sample) leads to less between-sample variation in expression than observed when comparing real bulk RNA samples, and argue that this is because in real samples there is systematic between-sample variation that is lost in the simplistic approach; by defining a "heterogeneous" pseudobulk strategy that tries to maintain inter-individual variation, they can generate pseudobulk samples that at least show more realistic levels of variation between samples. They conclude by demonstrating on a handful of deconvolution methods that their pseudo-bulk samples are generally more difficult to deconvolve than the simplistically-simulated samples.

Overall, the argument made by the authors is valid, and their experiments demonstrate it can make a difference in benchmarking. However, it seems fairly obvious even before seeing the results that the simplistic simulation strategy they compare against will yield reduced variation. In other words, the observation that there can be significant inter-individual differences in expression of the same cell type is pretty obvious to anyone working in the single cell field; it naturally follows that simulations should seek to maintain these differences where possible. Isn't it possible that the reason why the proposed simulation scheme is not more widely used currently is because there aren't that many datasets for which you have many individuals deeply sequenced across multiple cell types? For example, if a scRNA-seq dataset (that could be used to generate pseudobulk samples) only contains 10 individuals, then each simulated pseudo-bulk can only have cells from one of 10 original individuals. The authors' scheme tries to get around this point by allowing single simulated mixtures to contain contributions from multiple individuals (each cell type individually can only have contributions from one individual, but since the pseudo-bulk has more than one cell type, they can still mix from multiple individuals). But I think this is probably the primary reason why people haven't explored this approach previously (and not because they weren't aware of the effects of inter-individual variation).

- I appreciate that the main point of the manuscript is to demonstrate the value of the proposed simulation scheme for benchmarking pseudo-bulk samples, and the authors seem to want to try to not present "just another benchmarking paper". But the main contributions of the paper really come down to (1) showing the new framework generates variability closer to real samples than previous frameworks, and (2) showing some impact on the benchmarking. While Figures 1 and 3 really cover showing improved variation properties, the benchmarking experiment of Figure 5 is fairly shallow, particularly given how many deconvolution methods there are, and how many benchmarking papers have already been published recently. In my opinion, there are too many figures dedicated to just schematics (Figures 2 and 4). Given how mature deconvolution seems to be, the paper would benefit from much deeper benchmarking of the many more types of deconvolution methods that have been developed, and more demonstration that the new simulation framework yields changes in the conclusions of the benchmarking. For example, picking one of the recent benchmarking papers the authors cited, re-doing the benchmarking, and showing the conclusion is drastically different would be convincing.

- Part of what dampens my enthusiasm for this paper is that doesn't provide an actual framework for readers to use. For example, for methods to simulate scRNA-seq datasets, manuscripts in that area provide software that help readers actually generate better datasets (e.g. see scDesign2, also published in Genome Biology, https://genomebiology.biomedcentral.com/articles/10.1186/s13059-021-02367-2), as opposed to just discussing how best to generate datasets. Similarly here, I would expect the authors to provide actual software, example scRNA-seq datasets (such as those they use in the manuscript), and code to perform similar benchmarking and quantification of variability, etc., that the authors do in their manuscript.

- Simulated datasets often get criticized heavily because the simulation frameworks don't accurately capture all the sources of variability that real datasets have; this is something the authors acknowledge. Simulations have a place in benchmarking, not just because they have known ground truths that can be used for evaluation, but they can also be used to gain insight into those hidden confounders that drive variability in expression. In the scDesign2 example above, it is possible for example to fit scDesign2 to a dataset, sample from scDesign2, then compare the simulated to real data to see how close they are (and then potentially explore how the simulation can be revised to account for new factors of variation and see whether it improves the realism of the simulation). Related to the point above, making a framework available and showing how it could be used to explore sources of variation that drive inter-sample heterogeneity would be useful.

**Reviewer 2**

The authors proposed a new framework to simulate pseudobulk datasets for cell-type de-

convolution. Compared to the previous simulation pipeline, this framework can better preserve

gene variations among samples, which are more realistic to existing bulk samples. The authors

applied the proposed frameworks to benchmark several deconvolution algorithms and showed

that BayesPrism delivers the best performance while reference-free methods suffered the most.

This is an interesting work, but several important issues need to be addressed. These can be

summarized as follows:

Major concerns:

• In Figures 1 a and e, the results look like a mixture of batch effects and biological varia-

tions. Have the authors performed data integration before performing the t-SNE? Also,

from the Methods part, the scRNAseqa data are normalized using CPM, which is un-

common in single-cell data analyses. Can the authors provide more justifications for the

choice of this normalization method?

• I am confused about the steps of the proposed pipeline when I compared Fig 2 with the

Method section. From Fig 2, we will first generate n cell-type specific expression m × k

matrices, multiplied by the cell-type proportions matrix F . However, from the method

section (homogeneous simulation), how we will generate the Ck is unclear. Can the authors

provide more details?

• For the cell-type frequencies simulation, the authors proposed to utilize beta distribution,

which is a very interesting idea. However, if we use beta distribution, we need to generate

frequencies for each cell type and then normalize them to have a sum of 1. Can we directly

use Dirichlet distribution instead? Is there any specific benefit of Beta distribution?

• The authors have acknowledged the one drawback of this proposed framework is the loss of

dependencies between cell types. In addition, the framework will complicate batch effects

structure for the simulated psudobulk samples since different cell types have different

batch effects. How will this batch effect affect the downstream performances?

Minor concerns

• For Figure 1c, can the author also provide variances for the upper panel?

**Authors’ response**

Reviewer 1

**“it seems fairly obvious even before seeing the results that the simplistic simulation strategy they compare against will yield reduced variation.”**

We acknowledge that employing a simplistic simulation strategy can result in reduced variation. However, it is valuable to compare these methods to highlight their disregard for biological variations, which can result in deconvolution approaches producing overly idealized results when applied to bulk samples generated using such simulations. Our emphasis lies in demonstrating the unrealistic nature of these simulations, as they solely consider cell-type proportion variation while completely ignoring any biological variability. Consequently, benchmarking studies that have relied on such simulations may introduce bias into their findings.

**‘the observation that there can be significant inter-individual differences in expression of the same cell type is pretty obvious to anyone working in the single cell field’**

We agree that inter-individual differences is a well-acknowledged characteristic for single cell expression. However, our manuscript is not focused on characterizing these but is rather simply taking advantage of these differences to create realistic benchmarking methods. As we seek to find a realistic simulation that incorporates biological variability for more reasonable benchmarking, we are taking the advantage of such inter-individual differences in the ‘heterogeneous’ simulation we proposed.

**Isn't it possible that the reason why the proposed simulation scheme is not more widely used currently is because there aren't that many datasets for which you have many individuals deeply sequenced across multiple cell types**

It may be intuitive that the ‘heterogeneous’ simulation we proposed can only make at most n^k^ numbers of combinations (where n is the number of unique biological samples and k is the number of distinct cell types) of the cells, with each cellular component within a bulk sample originates from the entirety of cells derived from the same biological sample (let’s call it cell community for simplicity). For example, if there’s only 5 biological samples and 2 cell-types, this will only yield 25 different combinations.

However, in the real application of ‘heterogeneous’ simulation, additionally randomness is added by sub-sampling from such a cell community. This will lead to a number of possible combinations far exceeding n^k^. Specifically, we have the ‘use_chunk = ‘random’’ parameter to control that only a random proportion of single cells from the cell community will be used to construct a simulated bulk sample. This trick of sub-sampling from the same cell community has also been employed by some deep-learning based methods, which require massive amounts of training samples

(https://github.com/KevinMenden/scaden).

**if a scRNA-seq dataset (that could be used to generate pseudobulk samples) only contains 10 individuals, then each simulated pseudo-bulk can only have cells from one of 10 original individuals**

In addition to the sub-sampling strategies we clarified in the previous section for the 'heterogeneous' simulation to enhance randomness in the simulated bulk samples, in the revised version, we have introduced another 'heterogeneous' simulation called ‘heter-sampleIDfree’.' This new approach

completely eliminates the reliance on scRNA sampleID information and generates simulated samples with highly comparable biological variance as well. Specifically, it utilizes the sub-cluster information of each cell type and controls that each cell-type component in the simulated bulk sample is constrained to originating from the same sub-cluster. In the revised version, we demonstrated that deconvolution methods applied to bulk data simulated using the 'heter-sampleIDfree' approach also exhibit vulnerability due to the introduction of biological variance, beyond solely cell-type proportion variance. While the performance of all methods decreases from ‘homogeneous’ to 'heter-sampleIDfree', some methods are more robust to the heterogeneity change, which is aligned with our previous finding.

Fig S14. Comparison of regression-based methods: Line-plot comparing regression models

**The authors' scheme tries to get around this point by allowing single simulated mixtures to contain contributions from multiple individuals (each cell type individually can only have contributions from one individual, but since the pseudo-bulk has more than one cell type, they can still mix from multiple individuals)**

We acknowledge the comment and would like to clarify the purpose of introducing the threshold parameter in our previous submission. It is not aimed at increasing biological variance in the simulated samples; rather, it serves to ensure the quality of the cell community aggregation process. For instance, if we set a very low threshold (e.g., only 1 cell required to build a cell community), there might be missing reads from that specific cell, necessitating more cells to achieve a reasonable cell community profile.

Moreover, as techniques have become more mature, it is uncommon to obtain only a small number of cells per sample (. Consequently, aggregating over multiple samples (when the threshold is not met) is also infrequent. Depending on the number of cells obtained from single-cell sequencing, we recommend

setting the threshold close to the average number of cells per cell type. In such cases, it is generally not likely to create mixture signals from multiple biological samples for a particular cell type.

Reviewer 2

**the benchmarking experiment of Figure 5 is fairly shallow, particularly given how many deconvolution methods there are, and how many benchmarking papers have already been published recently; there are too many figures dedicated to just schematics (Figures 2 and 4)** We acknowledge this comment and have modified our figure layout, aiming for a more thorough comparison of the deconvolution methods. Schematic plots have now been moved to the supplementary figures.

**Given how mature deconvolution seems to be, the paper would benefit from much deeper benchmarking of the many more types of deconvolution methods that have been developed, and more demonstration that the new simulation framework yields changes in the conclusions of the benchmarking. For example, picking one of the recent benchmarking papers the authors cited, re-doing the benchmarking, and showing the conclusion is drastically different would be convincing.**

We acknowledge this comment and have included more deconvolution methods in the benchmarking. Specifically, in the revised version, we have included:

● for regression-based method: MuSiC, wRLM, RPC and Cibersort

● for marker-based methods: TOAST

We initially relied on the observations from the previously published benchmarking work Avila et al (cite https://doi.org/10.1038/s41467-020-19015-1) that the regression method matters very little while gene selection matters a lot and hence tested the simplest top performing regression method ‘nnls’ . However, we realized that this result too may be biased by the homogeneous simulation setting and we have now included other regression methods that use weights (MuSiC, wRLM) and more robust objectives (wRLM, RPC, Cibersort). Our latest results indicate that in the heterogeneous setting the choice of regression method does matter.

We have sourced the bulk simulation method used by Avila et al in our newly published benchmarking tool (https://github.com/humengying0907/deconvBenchmarking, with their simulation method named as ‘favilaco’). Visual assessment of the realism of bulk simulation methods concerning biological variance, pairwise similarity, and gene correlations indicates that their method lacks appropriate biological variance. This disparity explains the difference in our conclusions.

**Part of what dampens my enthusiasm for this paper is that doesn't provide an actual framework for readers to use. For example, for methods to simulate scRNA-seq datasets, manuscripts in that area provide software that help readers actually generate better datasets; I would expect the authors to provide actual software, example scRNA-seq datasets (such as those they use in the manuscript), and code to perform similar benchmarking and quantification of variability, etc., that the authors do in their manuscript; In the scDesign2 example above, it is possible for example to fit scDesign2 to a dataset, sample from scDesign2, then compare the simulated to real data to see how close they are (and then potentially explore how the simulation can be revised to account for new factors of variation and see whether it improves the realism of the simulation). Related to the point**

**above, making a framework available and showing how it could be used to explore sources of variation that drive inter-sample heterogeneity would be useful.**

We appreciate this feedback and have developed an R package **deconvBenchmarking** that includes all the functions mentioned above: https://github.com/humengying0907/deconvBenchmarking Specifically, deconvBenchmarking provides the following key features that allow for easy and efficient deconvolution benchmarking:

● Bulk Simulation Collection: a curated collection of bulk simulation methods with varying underlying heterogeneity levels to closely mirror real biological variability, as well as simulation methods from previously published benchmarking works (Avila Cobos et al. 2020, Sturm et al. 2019), and another methods published in the SCDC package. Additionally, the package comes with a set of visualization tools to assess the realism of the bulk simulations in terms of biological variance, pairwise similarity and gene correlations

● Built-in Deconvolution Methods: The package incorporates a diverse selection of pre-implemented deconvolution methods, thoughtfully categorized into four distinct groups: reference-free, regression-based, marker-based, and Bayesian methods. Researchers can easily apply and compare these methods on the simulated bulk data.

● Built-in reference construction: The package provides a collection of reference construction methods to create necessary input for regression-based and marker-based methods, which allows for combined evaluation of both deconvolution methods and reference construction methods.

● User-Friendly Framework: deconvBenchmarking offers an intuitive and user-friendly benchmarking framework for conducting deconvolution benchmarking experiments. It also offers the flexibility to include user-provided references and deconvolution methods for evaluation, allowing researchers to gain valuable insights into methods being tested.

● TCGA Expression Cohorts Integration: The package also offers options to include TCGA expression cohorts as an additional dataset for deconvolution evaluation. Users can directly compare the pre-calculated purity estimates with the results obtained from deconvolution analyses, allowing for assessment of deconvolution methods in the context of real-world data from large-scale cancer studies.

With the features listed above, users can easily reproduce the results we generated, explore different single cell inputs, and apply the framework to other deconvolution methods of interest. We also offered a tutorial for detailed implementation of the benchmarking framework and bulk simulation using an example dataset here:

https://humengying0907.github.io/deconvBenchmarking_tutorial.html

**2^nd^ round**

**Reviewer 1**

I appreciate the authors have performed a number of additional experiments to address the reviewers' comments. It is relatively clear that the proposed simulation scheme yields higher variance in the performance of the tested methods across categories, compared to the simplistic simulation scenario. The authors have also nicely released a github repo with code to make their framework more useful to the broader community. Given the additional experiments however, it is still not convincing that the conclusions would change with the new simulation strategy, or at least the visualization of the results needs to change to highlight the difference in conclusion.

For example, the abstract highlights BayesPrism and now MuSiC in the revised version. Looking at Figure 5 that compares the simulation strategies across all categories of deconvolution methods, it is clear the 'heter' simulations yield higher variance overall in performance of the methods in Figure 5a. However, at least in the visualization of Figure 5a, it looks like in the 'homo' simulation, MuSiC and BayesPrism consistently seem to be overall ranked highest, though there seems to be some ties in HNSCC. In contrast, in the 'heter' simulations, BayesPrism seems to be more poorly performing for 'melanoma cohort 1' and 'melanoma cohort 2' compared to at least one other method.

Similarly, in Figure 5b, BayesPrism and MuSiC seem to consistently achieve lowest RMSE in the 'homo' simulations, similar to 'heter'. The two curves (red/blue) seem correlated with each other, with the exception of the last method (CAMfree).


Zoomed in on specific classes of methods for Figures 3 and 4, the differences between homo and heter are mixed. Figure 3b shows the homo simulation poorly separates the methods, but it's not clear from the figure if the ranking is still similar. Figure 3c shows the comparison of reference choices vs regression choices is the same for heter and hom. There's too much overplotting in Figure 3d to tell if the conclusion of which approach is better is similar across hom and heter. The same goes for Figure 4a (too much overplotting).

Overall, I think the authors are doing a satisfactory job benchmarking against a broader set of methods with their new framework, but it's still unclear if the conclusions materially change, at least with respect to Figure 5 which arguably is what the conclusion (MuSiC and BayesPrism) is based on.

**Reviewer 2**

Thanks to the authors for providing a detailed explanation of my previous concerns. They

have answered some of my questions, but I still have some extra questions which would like

more details.

Major concerns:

• In Fig S2, it is unclear how to get the expression profiles C in Step (4) from Step (3).

Also, in Fig S2, figures in Step (4) seem to be a tensor, but in the text, it says C ∈ Rm×n.

• In the proposed heterogeneous framework, for a given cell type (say T cell) in a patient

(say patient 10), we use counts from all the T cells in patient 10. Namely, if simulated

samples i and j all contain the patient10’s T-cells, they will have the same column (T

cell) in step 4. This proposed framework only considers the mean of T cells within patient

10, ignoring the variance and randomness of those T cells.

• In Figure 1d, the authors selected the top 300 most variable genes from the baseline

expression. However, those genes may not necessarily be the most relevant genes. Those

genes may not include marker genes for rare cell types.

• The proposed pipeline will combine cell-types from different patients within one pseudo-

bulk sample, destroying the cell-cell interaction signals. Can the authors provide some

justifications on this impact since many marker genes are also receptor on the cell surface.

Minor concerns

• Fig S9 a, I will recommend the legend to be patient 1, patient 2, instead of tumor1,

tumor2. Becasue using tumor, it tends to imply those are tumor cells.

**Authors’ response**

Reviewer #1:

**I appreciate the authors have performed a number of additional experiments to address the reviewers' comments. It is relatively clear that the proposed simulation scheme yields higher variance in the performance of the tested methods across categories, compared to the simplistic simulation scenario. The authors have also nicely released a github repo with code to make their framework more useful to the broader community. Given the additional experiments however, it is still not convincing that the conclusions would change with the new simulation strategy, or at least the visualization of the results needs to change to highlight the difference in conclusion.**

**For example, the abstract highlights BayesPrism and now MuSiC in the revised version. Looking at Figure 5 that compares the simulation strategies across all categories of deconvolution methods, it is clear the 'heter' simulations yield higher variance overall in performance of the methods in Figure 5a. However, at least in the visualization of Figure 5a, it looks like in the 'homo' simulation, MuSiC and BayesPrism consistently seem to be overall ranked highest, though there seems to be some ties in HNSCC. In contrast, in the 'heter' simulations, BayesPrism seems to be more poorly performing for 'melanoma cohort 1' and 'melanoma cohort 2' compared to at least one other method. Similarly, in Figure 5b, BayesPrism and MuSiC seem to consistently achieve lowest RMSE in the 'homo' simulations, similar to 'heter'. The two curves (red/blue) seem correlated with each other, with the exception of the last method (CAMfree).**

We appreciate this valuable feedback and agree that our choices for visualization in the previous submission did not effectively highlight the contribution of our approach. Indeed, viewing the results from the perspective of relative ranking of the methods is more informative. In our revised version, we have generated new plots focused on ranks. We also agree that the melanoma cohorts I (labeled as Tirosh2016_SKCM in revised version) appear to be inconsistent with the others. To address this we have expanded our cohort collection to eight.

In our revised manuscript, we have aggregated the results into a ranking comparison plot reproduced below (revised Fig5a). Focusing on the rank based comparison, we did observe some reproducible patterns. Specifically, across the dataset being tested, BayesPrism and MuSiC (with CIBERSORTx based reference) maintained stable or superior rankings across different simulation scenarios, particularly in heterogeneous settings. Conversely, certain methods (reference free methods in particular) demonstrated a decline in ranking under heterogeneous simulation settings.

This observation highlights the inherent risks in evaluating methods purely on their performance in homogeneous environments, which might privilege methods optimized in simpler, low-variability contexts. We have modified our manuscript to include a comprehensive explanation on the ranking comparison plot. Moreover, we noted the underperformance of BayesPrism on Tirosh2016_SKCM is indeed an outlier. As we included more datasets in our benchmarking, BayesPrism ranked the 1st in the heterogeneous setting in 5 out of 8 datasets and was in the top 3 in another 2. Moreover, as we visualized the quantitative correlation on Tirosh2016_SKCM, the low performance of BayesPrism is less remarkable as it is quantitatively similar to other top performing methods.

Fig5a in the revised manuscript

We also provided the same plot (homo vs heter comparison) in terms of averaged Pearson correlation values (revised Fig5b) and RMSE values (revised FigS14). These visualizations offer insights into performance shifts across different simulation scenarios and emphasize our main point that a heterogeneous simulation prompts more reasonable benchmarking of deconvolution methods.

**Zoomed in on specific classes of methods for Figures 3 and 4, the differences between homo and heter are mixed. Figure 3b shows the homo simulation poorly separates the methods, but it's not clear from the figure if the ranking is still similar.**

We agree that our previous figures did not offer proper comparison of methods under homogeneous and heterogeneous settings. In the revised manuscript, we have updated Fig 3 and Fig 4 to incorporate detailed within-category comparisons using line plots, enabling a clearer understanding of method performance under different conditions. Specifically, the intersections between lines from the line plots indicates different rankings under different simulations. Furthermore, in Fig 5a, we have provided an explicit rank comparison across different method categories, enabling direct comparison of performance shifts under varying simulation scenarios. We have modified the manuscript to more accurately interpret our results. The previously un-separable performance under homogeneous conditions is now more clearly delineated in a bar plot that specifically illustrates the effect sizes associated with different methodological choices in these settings (Fig 3c).

Fig 3b in the revised manuscript

Fig 4a in the revised manuscript

**Figure 3c shows the comparison of reference choices vs regression choices is the same for heter and hom.**

We have revised this figure (revised Fig 3c) to show the impact of different methodological choices on the performance, incorporating all 8 datasets being evaluated. In this analysis, we fit a multi-linear regression to the Pearson correlation results with dataset and cell-type as a covariate, "nnls" as the baseline regression choice, and "no selection" with MuSiC regression as the baseline reference choice for comparison. The figure also shows 95% confidence intervals for the estimated effect size.

From the perspective of ranks, the heterogeneous setting changes the ranking of reference construction methods but not the ranking of regression methods. We also would like to note that even if ranks are not altered the heterogeneous setting emphasizes the effects of both methodological choices and thus provides a useful framework for further testing and method development. Specifically, in the homogenous setting the reference choice method contributes relatively little (0.0067 to .016) and the coefficients are *not significantly different*, though autogeneS slightly leads. However, within heterogeneous settings, the choice of reference method becomes significantly more impactful, contributing to correlation increases between 0.045 and 0.087. In this scenario, CIBEROSRTx dominates, while autogeneS is no better than simple differential expression-based methods.

Regarding regression method choices, although their relative rankings remain stable, the effect sizes become significantly more pronounced in heterogeneous settings. This underscores the suitability of MuSiC regression in heterogeneous environment. We have amended the text to more accurately reflect this summary.

Fig3c in the revised manuscript

**There's too much overplotting in Figure 3d to tell if the conclusion of which approach is better is similar across hom and heter. The same goes for Figure 4a (too much overplotting).**

We agree with the reviewer that the complex visualization does not emphasize key takeaway points. We have substantially revised and simplified the plots.

**Overall, I think the authors are doing a satisfactory job benchmarking against a broader set of methods with their new framework, but it's still unclear if the conclusions materially change, at least with respect to Figure 5 which arguably is what the conclusion (MuSiC and BayesPrism) is based on.**

While we provided specific recommendations regarding which methods perform best, as a simple take away message from our manuscript we hope that our contribution goes beyond that. Specifically, we are aiming to make a more general point that realistic simulations are necessary for benchmarking and that we can learn general principles about making reliable deconvolution methods. Besides the final ranking we highlight these contributions

-We provide Realistic performance estimates. Naive simulations often give a Pearson correlation of 0.99 while we get estimates that are more in line with that observed in practice (e.g. when parallel proportion measurements are available).

-Performance of reference free methods is poor in the heterogeneous setting demonstrating that these are unsuitable in most cases. Reference free methods continue to be developed and we show that it is critical to test them in realistic settings.

-Differences between regression and reference construction methods are much more pronounced in the heterogeneous setting highlighting the usefulness of the heterogeneous simulation framework for method development and testing.

-To facilitate further research, we have made our heterogeneous simulations easily accessible through user-friendly software compatible with any single-cell dataset. This contribution, we believe, adds substantial value to the field.

We have changed the text to more accurately reflect this emphasis.

Reviewer #2:

**Thanks to the authors for providing a detailed explanation of my previous concerns. They have answered some of my questions, but I still have some extra questions which would like more details. In Fig S2, it is unclear how to get the expression profiles $C$ in Step (4) from Step (3). Also, in Fig S2, figures in Step (4) seem to be a tensor, but in the text, it says $C \in \mathbb{R}^{m \times n}$.**

Thank you for bringing this to our attention. The cell-type specific expression profiles C should have dimension \mathbb{R}^{m \times k}, which is a gene by cell type 2-d matrix indicating cell-type specific profiles for each individual and will be weighted by simulated fraction subsequently to represent one simulated sample. This has now been corrected.

*The tensor-like visualization in figS2 step(4) represents the cell-type specific expression profiles* ***for all*** *n samples, which can be viewed as a combination of n different cell-type specific expression profiles C, where each individual profile C contains the information as illustrated above.*

*To generate cell-type specific profiles C from step(3) to step(4), we take the average expression values for all single cells from the same cell type. For example, in step(3), all the single cells in “combo1” will be used to generate simulated-sample1. By taking the average expression profiles of single cells from each cell type in ‘combo1’, we get a \mathbb{R}^{m \times k} cell-type specific expression profiles C for this simulated-sample1. We then repeat the process across n simulated samples to get all the cell-type specific expression profiles for each simulated sample.*

We have modified the legend for fig S2 to make this more clear.

**In the proposed heterogeneous framework, for a given cell type (say T cell) in a patient (say patient 10), we use counts from all the T cells in patient 10. Namely, if simulated samples $i$ and $j$ all contain the patient10's T-cells, they will have the same column (T cell) in step 4. This proposed framework only considers the mean of T cells within patient 10, ignoring the variance and randomness of those T cells.**

Thank you for your valuable comment regarding the handling of cell type variance in our proposed heterogeneous framework. We realize that our initial submission did not clearly explain how our simulation pipeline specifically addresses this issue. After the “decision” to use patient 10’ T cells as the T cell components for a simulated sample, an additional randomization is applied to these cells. Specifically, we will randomly select between 50-100% of the single cells from patient 10’ T cells for aggregation, which prevents using the exact same cells across different simulated samples. We have amended our method selection to include this aspect.

**In Figure 1d, the authors selected the top 300 most variable genes from the baseline expression. However, those genes may not necessarily be the most relevant genes. Those genes may not include marker genes for rare cell types.**

We appreciate this valuable feedback and have extended the comparison to cell type specific genes as well (revised Fig S4). Under both settings, the gene correlation is better preserved with heterogeneous simulation, whereas homogeneous-simulated samples exhibit spuriously high (or low) gene correlations. Below we provided an example of pairwise correlations for all marker genes (derived from limma-based DE analysis) from the melanoma cohort II, the heter-simulated samples largely preserved the gene correlation structures as presented in the real bulk samples (labeled as baseline).

Fig S4 in the revised manuscript

**The proposed pipeline will combine cell-types from different patients within one pseudo-bulk sample, destroying the cell-cell interaction signals. Can the authors provide some justifications on this impact since many marker genes are also receptor on the cell surface.**

We agree this is a limitation of this approach and this is discussed in the text. We think that this is a reasonable choice for several reasons. Firstly, a simulation that would capture this kind of relationship would first need to estimate the ground truth covariance and would require a large cohort to do so accurately. Secondly, we do not believe that this deviation from real data should significantly impact the benchmarking results. While proportion estimation accuracy of a specific cell type X depends on the absolute cell fraction of X, it is less sensitive to the fractions of other cell-types.

The potential problem would be most acute for cell-types whose expression profiles are very close. If the two very similar cell-types are also correlated in proportion (beyond the trivial dependence induced by enforcing that proportions sum to 1) the proportion estimation problem becomes more difficult in a way that is not captured by our framework. However, we believe this to be a relatively minor effect considering that we test proportion estimation for cell types that are sufficiently distinct.

**Fig S9 a, I will recommend the legend to be patient 1, patient 2, instead of tumor1, tumor2. Because using tumor, it tends to imply those are tumor cells.**

We appreciate this feedback and have modified the legend accordingly.

**3^rd^ round**

**Reviewer 2**

Thanks to the authors for addressing my previous concerns. They have solved many of the

concerns but I still have some extra questions which would like more details.

Major concerns:

• For the step (4) Fig S2, it is better to denote Ci ∈ Rm×n as the ith expression profile.

The caption of step (4) should also be modified.

• From another benchmark paper, “Benchmarking of cell type deconvolution pipelines for

transcriptomics data”, FARDEEP (Fast and Robust Deconvolution of Tumor Infiltrating

Lymphocyte from Expression Profiles using Least Trimmed Squares) is one of the best

performed model. Unlike other regression-based model, FARDEEP uses least trimmed

squred framework. It will be interesting to see how FARDEEP performed under different

setting.

• The results in Figure 3C is kind of confusing. Can the authors provide more details

for the multi-linear regression model? Also for completeness, it is better also include

semi-heterogeneous simulation results.

• In Figure 4b, debCAM showed very high variance for the RMSE in multiple datasets

but had quite consistent result for pearson correlation. Can the authors provided some

explaination on this? Also, I couldn’t find xCell’s results in Fig 4b as the authors claimed

“ ...observed that xCell signatures effectively predicted the true abundance within each

cell type (Fig 4b, Additional file 1: Fig S13)”.

Minor concerns

• In the Result section, the authors claimed “We proposed a “heterogeneous” simulation

setting where single cells used to constitute a simulated bulk sample are constrained to

come from the same biological samples, leveraging the sample-level heterogeneity from

the single-cell data”. It is inaccurate for this claim since different cells from different cell

types may drawn from different samples.

• CIBERSORT/CITERSORTX should be all capitalized. There are multiple place where

the authors used Cibersort, cibersort, and CibersortX. Please be consistent.

**Authors’ response**

1) Regarding the suggestions to use C_i in Supplementary Fig S2, we have modified related figures and figure legend accordingly.

2) Regarding the suggestions to include FARDEEP in the comparison, we have conducted additional experiments to include the FARDEEP method in the comparison, and tested the performance of it throughout all the experimental repeats and dataset being used. In the revised Supplementary Fig. 11, we showed that this regression- based method, which utilizes adaptive least trimmed squares in its optimization, demonstrates similar results compared to other robust-regression based methods like wRLM and RPC. In particular, it demonstrated comparable sensitivity to heterogeneity change in the bulk sample, lagging behind the top performer MuSiC under heterogeneous simulation setting. This comparison further consolidated our previous finding that robust-regression based regression tends to behave similarly; the results are also included in our revised manuscript.

3) Regarding the confusion of the multi-linear regression model in Fig 3c, we have included a separate Method section explaining the model being used in this analysis. We have also added the results from the semi-heterogeneous simulation model for completeness.

4) Regarding the high variance of RMSE value for debCAM in Fig 4b, we have included an explanation in the revised manuscript specifically targeting this observation:

“... we also observed that debCAM demonstrates more variation in the RMSE values across experimental repeats, and generally exhibits higher RMSE compared to regression-based methods. This suggests that while debCAM accurately captures correlations, it is less effective at capturing the correct magnitude of the cell type fractions. Indeed, for debCAM, the absolute fraction inference is performed as a post- hoc adjustment to the scores, therefore, it does not consistently yield accurate estimations.”

5) For the remaining comments from the reviewer, we highly appreciate these comments and have addressed the problems being identified. All figures have been clearly referenced throughout the manuscript, and we have ensured consistent capitalization of 'CIBERSORT' in both the text and figures. Note that we retained 'CIBERSORTx' as opposed to ‘CIBERSORTX’, in accordance with the nomenclature used in the original CIBERSORTx publication.
